# An updated estimation approach for SEIR models with stochastic perturbations: Application to COVID-19 data in Bogotá

**DOI:** 10.1371/journal.pone.0285624

**Published:** 2023-08-21

**Authors:** Andrés Ríos-Gutiérrez, Soledad Torres, Viswanathan Arunachalam

**Affiliations:** 1 Department of Statistics, Universidad Nacional de Colombia, Bogotá, Colombia; 2 School of Mathematics, Universidad Industrial de Santander, Bucaramanga, Colombia; 3 CIMFAV - Facultad de Ingeniería, Universidad de Valparaíso, Valparaíso, Chile; Texas Christian University, UNITED STATES

## Abstract

This paper studies the updated estimation method for estimating the transmission rate changes over time. The models for the population dynamics under SEIR epidemic models with stochastic perturbations are analysed the dynamics of the COVID-19 pandemic in Bogotá, Colombia. We performed computational experiments to interpret COVID-19 dynamics using actual data for the proposed models. We estimate the model parameters and updated their estimates for reported infected and recovered data.

## Introduction

**Fig 1:**
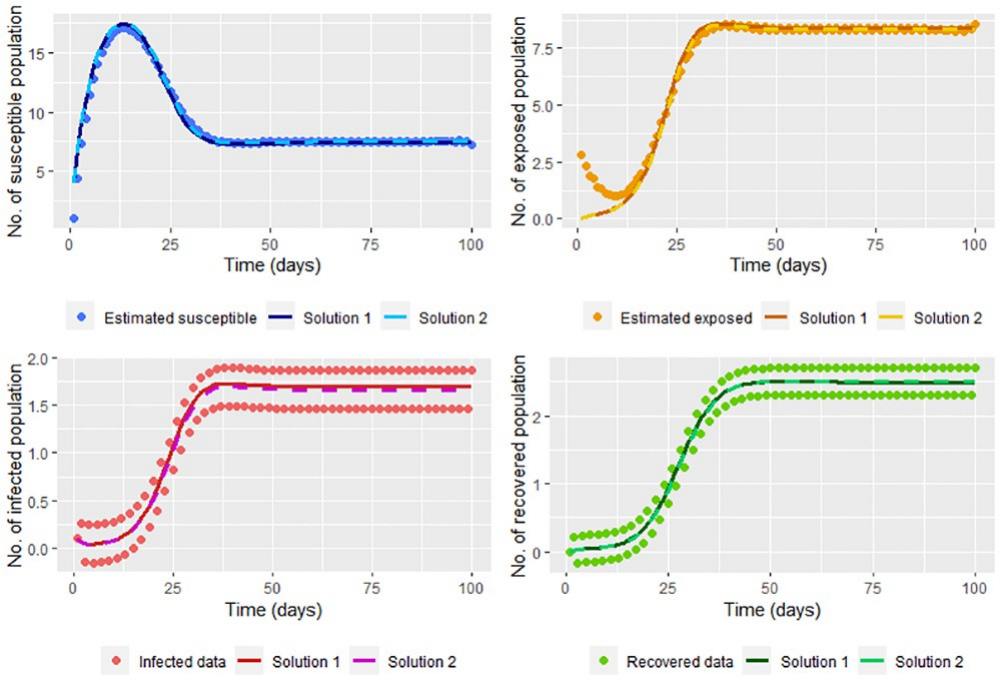
COVID-19 infected data from Bogota D.C. during the first 385 days.

The Latin American region is one of the regions that has reported more COVID-19 infection cases. At the end of the year, 2019, the World Health Organization (WHO) said the outbreak started in Wuhan, China. This outbreak quickly spread to more than 50 countries in one month. On January 30^th^/2020, the WHO declared the COVID-19 epidemic a Public Health Emergency of International Concern (PHEIC) [1]. European countries such as Spain, France, and Italy have had a significant number of deaths and a high number of infected cases. In March 6^th^/2020, the first case of COVID-19 coronavirus was confirmed in Colombia [2]. Colombia has implemented many emergency measures in response to the coronavirus outbreak, including strict lock-downs, PCR testing capacity, contact tracing, and augmenting ICU capacity in the hospitals. In particular, Colombia is one of the top ten countries globally regarding the registered number of infections for more than 2 million cases and more than 58,974 deaths since March 2020, regarding the data of the COVID-19 of the Instituto Nacional de Salud Colombia (INS) [3] (See Fig 1). In Colombia, national and local governments have taken measures to control the spread of infections, such as lockdowns, restrictions movements, including closing airports, etc. [4, 5]. However, there was still a rapid spread of the virus in Colombia, even when the vaccination in Colombia started on February 17^th^ 2021 and, in May 22^nd^ 2021 there were 9,325,861 vaccinated people. [6]

Given the conditions of contagion transmission and spread in the country, it is necessary to establish when the rise of cases is expected to be high and when the isolation and modality restriction measures can be repealed. To mention a few models, SIR (susceptible, infected, and recovered), SIS (susceptible and infected), and SEIR (susceptible, exposed, infected, and recovered). The first epidemiological mathematical model in 1927 by Kermack and McKendrick in [7], the SIR model. In this model, three types of individuals are distinguished: susceptible in the time *t* (*S*(*t*)), infected in the time *t* (*I*(*t*)), and removable in the time *t* (*R*(*t*)). Susceptible individuals are prone to contracting a disease by having infectious contact with these infected who may present symptoms. Removable individuals cease to be infected, or either die or have immunity against the disease.

In the SIR model, *β* is the *transmission rate*. Thus, *βI*(*t*)*S*(*t*) is the total of *S*(*t*) susceptible who acquire the infectious agent when having contact with *I*(*t*) infectious population on time *t*. 1/*γ* is the *recovered time* (*γ* is the *recovered rate*), that is, the time that takes an infected individual to recover in a removable individual. This way, *γI*(*t*) is removable of *I*(*t*) infected individuals on time *t*. Something intrinsic to the SIR model does not consider the *exposed population*, who are the individuals that have the infectious agent, but they can not spread the epidemic. The notation is *E*(*t*) as the number of exposed populations in the day *t*. That population can be the infection agent (virus, bacteria, etc.) but is not infectious until they have completed the entire incubation. This time is time that takes an individual to recover from a contagious individual, which we denote by 1/*υ*. In COVID-19, there is also an incubation time from 2 to 14 days [8]. For this reason, we consider a more general model: the SEIR model [9]. In the SEIR model, *βI*(*t*)*S*(*t*) and *γI*(*t*) are interpreted similarly to the SIR model. In this model, *υ* is the incubation rate. The SIR and SEIR models assume the total population is constant. For example, the SEIR model is

N(t)=S(t)+E(t)+I(t)+R(t)

The modified SEIR model is the *SEIR model with demographics*, where Λ represents the *influence rate*, that is, the average number of new susceptible populations per unit of time [10]. The *emigration rate* is denoted as *μ*, and *γ* is the *recovered rate*. Therefore, *γI*(*t*) is the total recovered of *I*(*t*) infected individuals on time *t*. The equation of the SEIR model with demographics is the ordinary differential system 1.

$$\begin{array}{c} \left\{ \begin{array}{cc} \frac{\begin{array}{c} dS(t) \end{array}}{\begin{array}{c} dt \end{array}}=\Lambda -\beta I\left(t\right)S\left(t\right)-\mu S\left(t\right) & \\ \frac{\begin{array}{c} dE(t) \end{array}}{\begin{array}{c} dt \end{array}}=\beta I\left(t\right)S\left(t\right)-\upsilon E\left(t\right)-\mu E\left(t\right) & \\ \frac{\begin{array}{c} dI(t) \end{array}}{\begin{array}{c} dt \end{array}}=\upsilon E\left(t\right)-\gamma I\left(t\right)-\mu I\left(t\right) & \\ \frac{\begin{array}{c} dR(t) \end{array}}{\begin{array}{c} dt \end{array}}=\gamma I\left(t\right)-\mu R\left(t\right) \end{array}\right. \end{array}$$ (1)

Recently, research articles have been studied using different approaches to model COVID-19, to mention a few [11–15]. They focus on minimizing the sum of squares based only on the infected population data in taking the parameters of other papers. Statistically, it should be used all the types of populations under an epidemic and a methodology to estimate the parameters of the model. Actually [16] is highlighted that “*it is difficult to consider all possible interactions between interventions in the same model and find parameters close to reality through simulations*”. In this way, we must search for a model which includes all the populations under the epidemic and estimates the parameters based on as much information as possible. In particular, to Bogotá (Colombia), there are previously published papers as [17–19] whose focus is taking the parameters under which a good fit is observed. These papers are based on SEIR models with types of population, for instance, asymptomatic or hospitalized individuals, but they do not estimate the susceptible and exposed population. However, the fit is only on the number of reported cases of COVID-19, leaving aside the recovered population, whose data is being reported by the [3]. The mean objective of this paper is to provide three different methods to estimate parameters on models based on epidemics, taking as an example the SEIR model: (i) minimizing the loss function considering infected and recovered data; (ii) using a data update approach; and (iii) using a stochastic infection rate. We also give a possible implementation to actual data, in this case, considering the COVID-19 data from Bogotá—one of the methods to estimate the susceptible and exposed population: using the data update estimation.

This paper describes methods for estimating SEIR-type models for COVID-19 data in Bogota. The paper is organized as follows: The section “A state of the art on the parameter estimation on epidemic models” proposes two models based on the minimization of loss functions based on infected population *I*(*t*) and recovered population *R*(*t*) data. The section on “Estimation of the SEIR MODEL from real data” is devoted to parameter estimation based on the susceptible S and exposed E population, which has been previously estimated using real data. Additionally, we update the parameters *β*, *υ*, and *γ* as a function of time. The section on “Estimation of the SEIR MODEL from real data” pres-ents an SEIR model with random perturbations, i.e., where we assume that the parameter *β* is random. We also include the corresponding parameter estimation method for real data for this model. Finally, the last section concludes the paper with future perspectives and the advan-tages and disadvantages of each of the proposed methodologies.

## A state of the art on the parameter estimation on epidemic models

### State-of-art

**Fig 2:**
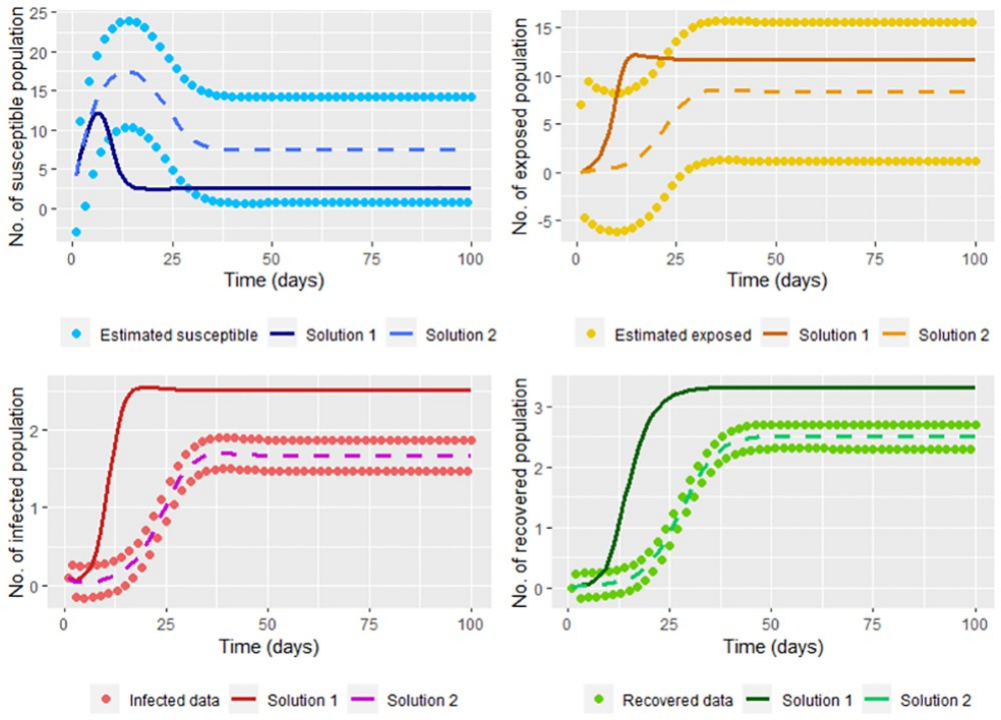
COVID-19 data from Bogota D.C. during of the first 385 days.

This paper establishes an estimation methodology that could improve the forecasts for the populations in an epidemic under the SEIR model. For example, we take COVID-19 data from Bogota during the first 385 days of the pandemic. According to the test, we assume that the number of infected populations is the number of individuals diagnosed as infected. The number recovered population is the number of individuals who, after being positive for COVID-19, had a negative test reported each day or who have been 14 days without symptoms. We do not consider the recovered data. The cumulative number of tests reported as negative measures individuals could be reported as newly infected in the following days when the patient retests once they present new symptoms. Data is taken from the public database of the INS [3]. In addition, we consider that the infection rate is not the same for all 385 days since most people from Bogota were initially isolated by control and prevention measures, which can be searched in [5]. After around 150 days, there were economic reactivation and Christmas holidays, so the infection rate differed (see Fig 2). We have **six periods** of time, each with different infection rates, which were chosen regarding to the rules and laws established by the Colombian Government and mayor’s office of Bogota. These points do not coincide necessarily with the inflection points of the infection function. We assume that the infection rate of each period changed according to the measures of mitigation and control against COVID-19 by the Bogota D.C. city [5]. Some of these points coincide with some peaks and valleys found in the graph of the smoothed infected data (see Fig 2). On the other hand, we do not consider variants of the disease, due to the available database does not have of the type of variant for each infected individual. The intervals are noted by *τ*_1_,…, *τ*_6_. In the Fig 2, we smoothed the data using the function frfast from npregfast package in R.

**Fig 3:**
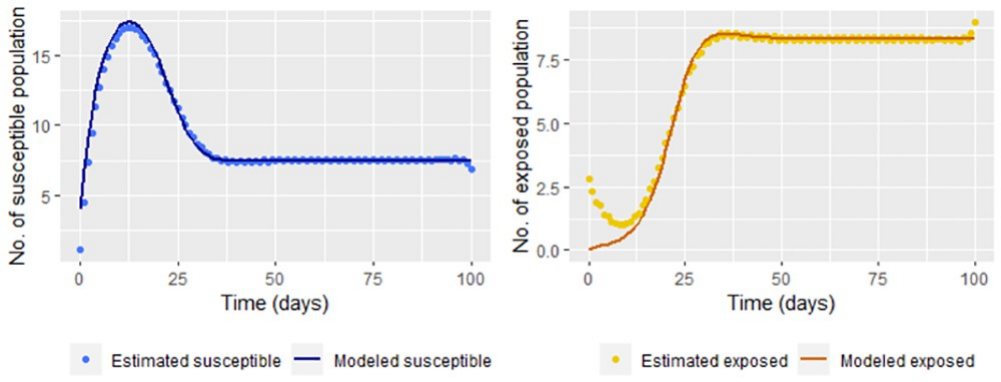
Estimated infected and recovered population of the 4 model with ${\hat{\delta }}_{1}^{2}=\sum_{j=1}^{n}(I(t_{j})-\phi_{3}(t_{j}){)}^{2}/n$ for each considered interval *I*_*r*_, *r* = 1, …, 6, using the mle2 function (parameters on the Table 1).

We propose to study the COVID-19 over-estimation methods using the system 1. There are different statistical methods of estimating parameters for the deterministic models, namely, a trial-and-error method, the use of computational algorithms for minimizing the sum of squares(see [20–25] or on the available data [26]). Also, it is worth mentioning that the papers [27–31] consider the parameters as a function of time *t* and estimate their values. However, some papers are based only on infected data, which drives possible flawed estimations on another type of population under an epidemic (Fig 3). Other papers, as [32, 33] suggest an overestimation of the infected population or even that the models with differential equations do not work to predict the infected population.

We use the maximum likelihood method to estimate the parameters using COVID-19 data from Bogota under the SEIR model with the regression model given by the Eq 2. We take Λ = (9.42/1000), $\bar{p}=$73,660 with $\bar{p}$ as the average of the projected population of Bogota from march 6^th^ of 2020 to march 21^th^ of 2021 according to DANE, which is the Official Statistical Office from Colombia, the birth Λ = 9.42/1000 and the death rate *μ* = 4/1000 (see [34]). We note *A*_*k*_(*t*) with *k* = 1, …, 4 as *S*(*t*), *E*(*t*), *I*(*t*) and *R*(*t*), respectively, on 2. In addition *ϕ*_*k*_ (*t*), *k* = 1, …, 4 is noted on 2 as the regression function which corresponds to the approximate solution of the system 1 for *S*(*t*), *E*(*t*), *I*(*t*) and *R*(*t*), respectively. We do not have the analytic solution of the system 1 on 2, therefore the approximate solution refers to the solution according to numerical methods according to ode function from deSolve package in R.

$$\begin{array}{c} A_{k}\left(t\right)=\phi_{k}(t)+\epsilon_{k}\text{,}\;\epsilon_{k}∼N\left(0,\delta^{2}\right). \end{array}$$ (2)

On 2 we propose to estimate *δ*^2^ as the variance given by

$$\begin{array}{c} {\hat{\delta }}^{2}=\sum_{k=1}^{4}\sum_{j=1}^{n}\frac{{\left(A_{k}\left(t_{j}\right)-\phi_{k}(t_{j})\right)}^{2}}{n}\text{,} \end{array}$$ (3)

thus, we use $A_{k}(t_{j})∼N(\phi_{k}\left(t_{j}\right),{\hat{\delta }}^{2}),j=1,...,n$ for the parameter estimation (see [35]). To this extent, we add noise for each of the variables *A*_*k*_(*t*_*j*_) with *k* = 1, …, 4 to make a maximum likelihood estimation. We wish to mention that the recent papers [21, 23, 25] fit the models only to the infected data. Following that focus and the notation *ϕ*_*k*_ (*t*_*j*_), *A*_*k*_ (*t*_*j*_), we re-write the model 2 as the equation given by

$$\begin{array}{c} \text{Model}\;\text{1}\text{:}\;A_{k}\left(t\right)=\phi_{k}(t)+\epsilon_{k}\text{,}\;\epsilon_{k}∼N\left(0,\delta_{1}^{2}\right). \end{array}$$ (4)

with ${\hat{\delta }}_{1}^{2}=\sum_{j=1}^{n}(I(t_{j})-\phi_{3}(t_{j}){)}^{2}/n$ (see Fig 3), which is a model with noise only for *I*(*t*). We use mle2 function from bbmle package from R, to establish the parameters for each period. We take six periods instead of 8 because intervals 2 and 3 and 4 and 5 do not have inflection points for the infected population. These parameters can be observed in the Table 1

**Table 1:** Estimated parameters of the 4 model with ${\hat{\delta }}_{1}^{2}=\sum_{j=1}^{n}(I(t_{j})-\phi_{3}(t_{j}){)}^{2}/n$, for each interval *I*_*r*_, *r* = 1, …, 6. We use the mle2 function.

Period of time (*τ*_*s*_)	$\beta_{\tau_{s}}{\text{days}}^{-1}$	$\upsilon_{\tau_{s}}{\text{days}}^{-1}$	$\gamma_{\tau_{s}}{\text{days}}^{-1}$
[0,150)	1.738857e-05	1.383661e-05	1.633080e-02
[150,205)	9.882397e-01	1.201782e-11	5.492948e-03
[205,270)	1.000000e+00	1.337070e-06	1.060467e-03
[270,312)	1.290321e-01	1.815878e-05	1.780064e-02
[312,363)	9.999918e-01	5.725818e-07	3.661433e-02
[363,385]	7.040308e-01	4.579072e-06	3.334993e-03

**Fig 4:**
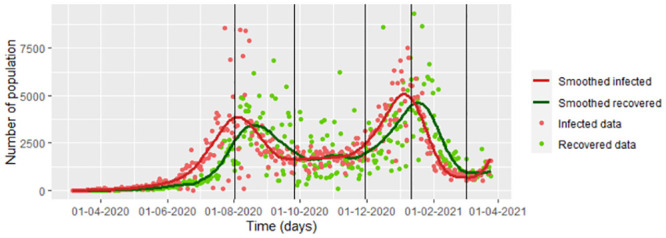
Susceptible and exposed estimation on the interval *I*_1_ = [0, 150), according to the model 4 with ${\hat{\delta }}_{1}^{2}=\sum_{j=1}^{n}(I(t_{j})-\phi_{3}(t_{j}){)}^{2}/n$.

Note in the Fig 3 that on the interval [312, 363), the recovered population is over-estimated. Other people could be deficiently estimated as is observed in Fig 4, whose graph is on the interval *I*_1_ = [1, 150) we have that the exposed population is a maximum 10,410,375, which is not valid since the people of Bogota and its metropolitan area is less than 9,000,000 (see [34]).

**Fig 5:**
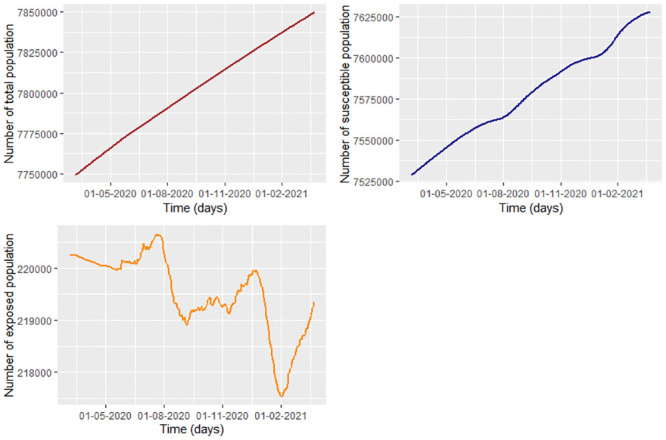
Negative log-likelihood function to *β* ∈ [0, 1], *υ* = 1.201782 × 10^−11^ and *γ* = 5.492948 × 10^−3^ on the interval [150, 205) for COVID-19 data from Bogota D.C.

On the other hand, when we graph the negative log-likelihood function on the interval [150, 205) for *β* with *β* ∈ [0, 1], *υ* = 1.201782 × 10^−11^ and *γ* = 5.492948 × 10^−3^ (Fig 5) note that this function does not have a monotone behavior. Therefore, if an initial value of *β* is near to a local minimum when the mle2 function is used since estimation can vary significantly. The estimation of *β* is necessary for the susceptible and exposed population data using the approximation of the Euler method as given in the Eq 8.

### Data modeling

Since we have recovered data for COVID-19 data from Bogota, we initially propose an estimation method based on that data re-writing the model 2, taking the notation given by *ϕ*_*k*_ (*t*_*j*_), *A*_*k*_ (*t*_*j*_), as

$$\begin{array}{c} \text{Model}\;\text{2}\text{:}\;A_{k}\left(t\right)=\phi_{k}(t)+\epsilon_{k}\text{,}\;\epsilon_{k}∼N\left(0,\delta_{2}^{2}\right). \end{array}$$ (5)

where $A_{k}(t_{j})∼N(\phi_{k}\left(t_{j}\right),{\hat{\delta }}_{2}^{2}),j=1,...,n$ with

$$\begin{array}{c} {\hat{\delta }}_{2}^{2}=\sum_{i=1}^{n}\frac{{\left(I\left(t_{i}\right)-\phi_{3}(t_{i})\right)}^{2}}{n}+\sum_{i=1}^{n}\frac{{\left(R\left(t_{i}\right)-\phi_{4}(t_{i})\right)}^{2}}{n}\text{.} \end{array}$$ (6)

**Fig 6:**
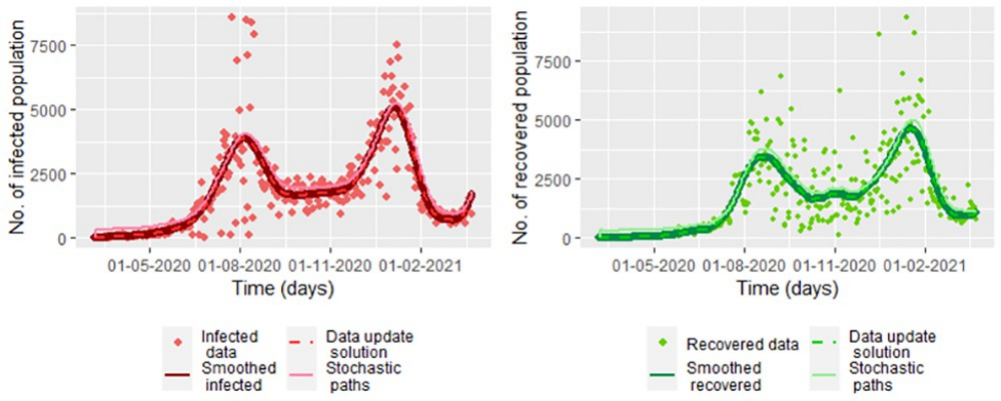
Estimated infected and recovered population of the 5 model with ${\hat{\delta }}_{2}^{2}$ given by the Eq 6 for each considered interval *I*_*s*_, *s* = 1, …, 8. We use the mle2 function (parameters on the Table 2).

**Fig 7:**
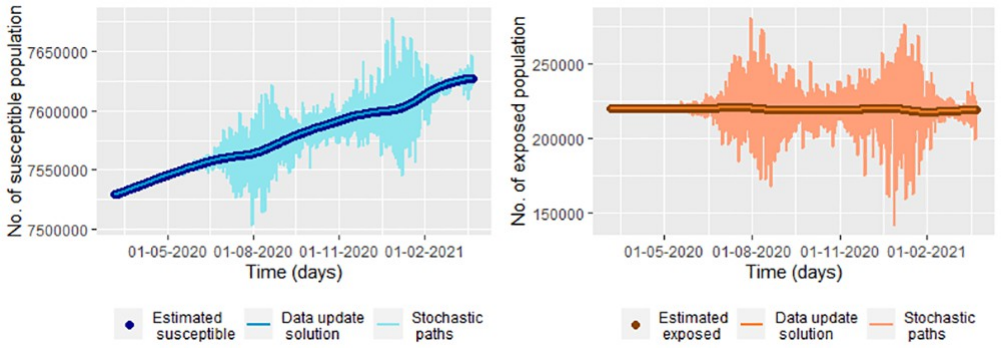
Susceptible and exposed estimation on the interval *I*_1_ = [1, 150) according to the model 5 with ${\hat{\delta }}_{2}^{2}$ given by the Eq 6.

Note that in the model 5, we have noise on the infected and recovered data, which is all the available data. We do not consider the possible correlation between the infected and recovered population to simplify the calculus. We observed the inflection points graphically for the recovered population, which can be seen in Fig 6. Based on these points, combined with the points with changes in control measures on the infected population regarding [4], we chose eight different intervals for which the parameters change, under the influence of the recovered data. In the Fig 6, we also can see the estimations of the model 5 with ${\hat{\delta }}_{2}^{2}$ given by the Eq 6. Note that the difference between Figs 4 and 7 is that the first one shows the estimation under the loss function uniquely considering the infected data (Model 1), while the second one shows the estimation under the loss function considering the infected data and the recovered data (Model 2). In this way, taking a loss function involving the two types of data improves the estimation of the recovered population.

**Table 2:** Estimated parameters of the 5 model with ${\hat{\delta }}_{2}^{2}$ given by 6, for each considered interval *I*_*s*_, *s* = 1, …, 8. We use the mle2 function.

Period of time (*τ*_*s*_)	$\beta_{\tau_{s}}$	$\upsilon_{\tau_{s}}$	$\gamma_{\tau_{s}}$
[0,150)	9.999e-01	2.523e-06	8.115e-03
[150,177)	5.415e-01	3.301e-06	1.500e-02
[177,205)	1.000e+00	7.454e-29	4.098e-19
[205,270)	1.000e+00	1.337e-06	1.060e-03
[270,312)	1.290e-01	1.816e-05	1.780e-02
[312,330)	9.817e-01	1.941e-15	1.039e-02
[330,363)	1.000e+00	1.000e-06	3.806e-19
[363,385]	7.040e-01	4.579e-06	3.335e-03

As we could note in the Fig 4, when we use the estimated model 5 with ${\hat{\delta }}_{2}^{2}$ given by 6, the susceptible and exposed population can be over-estimated or under-estimated. According to the estimations (see Fig 7), for day 150, there are 12,535,024 of the exposed population, which is not valid. It also can be seen that on day 150, the susceptible population is 38 approximately, something illogical since around day 300 there are more than 5,000 reported cases (see Fig 2).

Figs 4 and 7 made us think about studying an estimation method that also considers the susceptible and exposed population. For that, we propose initially a parametric method of estimation where first the exposed and susceptible population is estimated. After, we find the necessity of establishing a data update estimation, which we propose in the following subsection.

## Estimation of the SEIR model from real data

This section will propose an updated data method that searches for a good fit to avoid overestimating parameters such as recovery rate *γ*. It is not adequate to take only one interval since there are some different conditions of infection spread according to local government [5]. The model to be estimated by a theoretical approach is the Model 2 of the Eq 2.

### Parameter estimation on the SEIR model with susceptible and exposed missing data

We have the data corresponding to infected and recovered population per day from Bogota (Colombia), whereby the other data (susceptible and exposed population) are missing data. In this way, we will estimate the parameters using only the available data. If we think of the ordinary least squares method, we must minimize the equation

$$\begin{array}{c} Q=\sum_{i=0}^{n}{\left(I\left(t_{i}\right)-\hat{I}\left(t_{i}\right)\right)}^{2}+{\left(R\left(t_{i}\right)-\hat{R}\left(t_{i}\right)\right)}^{2}, \end{array}$$ (7)

where $\hat{I}$ and $\hat{R}$ are the solutions of the deterministic system 1. However, we do not have the analytical solution for *I* and *R* of the system 1, the reason why $\hat{I}$ and $\hat{R}$ are the approximations given by the Euler method (see [36]),

$$\begin{array}{c} \begin{array}{c} \hat{S}(t_{j})=S(t_{j-1})+[\Lambda -\beta S(t_{j-1})I(t_{j-1})-\mu S(t_{j-1})](t_{j}-t_{j-1})\\ \hat{E}(t_{j})=E(t_{j-1})+[\beta S(t_{j-1})I(t_{j-1})-\upsilon E(t_{j-1})-\mu E(t_{j-1})](t_{j}-t_{j-1})\\ \hat{I}(t_{j})=I(t_{j-1})+[\upsilon E(t_{j-1})-(\mu +\gamma )I(t_{j-1})](t_{j}-t_{j-1})\\ \hat{R}(t_{j})=R(t_{j-1})+[\gamma I(t_{j-1})-\mu R(t_{j-1})](t_{j}-t_{j-1}). \end{array} \end{array}$$ (8)

However, note in the Eq 8 that $\hat{I}\left(t_{j}\right)$ needs the values of *E*(*t*_*j*−1_) reason why we have to estimate *S*(*t*_*j*_) and *E*(*t*_*j*_). For it, first note that

$$\begin{array}{c} S(t_{j})+E(t_{j})=N\left(t_{j}\right)-I\left(t_{j}\right)-R\left(t_{j}\right)\text{,}\; \end{array}$$ (9)

where *N*(*t*_*j*_) is the total population in the time *t*_*j*_.

Note that,

$$\begin{array}{c} \hat{S}(t_{j})+\hat{E}(t_{j})=S(t_{j-1})+E(t_{j-1})+[\Lambda -\mu \left(S(t_{j-1})+E(t_{j-1})\right)-\upsilon E(t_{j-1})](t_{j}-t_{j-1}) \end{array}$$ (10)

Replacing *S*(*t*_*j*−1_) + *E*(*t*_*j*−1_) by *N*(*t*_*j*−1_) − *I*(*t*_*j*−1_)−*R*(*t*_*j*−1_) and $\hat{S}\left(t_{j}\right)+\hat{E}\left(t_{j}\right)$ by *N*(*t*_*j*_) − *I*(*t*_*j*_) − *R*(*t*_*j*_), to observe for *E*(*t*_*j*−1_) and *S*(*t*_*j*−1_) given by

$$\begin{array}{c} \begin{array}{cc} \overset{˘}{E}(t_{j-1})= & \frac{\begin{array}{c} N\left(t_{j-1}\right)-N\left(t_{j}\right)-I\left(t_{j-1}\right)+I\left(t_{j}\right)-R\left(t_{j-1}\right)+R\left(t_{j}\right) \end{array}}{\begin{array}{c} \upsilon (t_{j}-t_{j-1}) \end{array}}+\frac{\begin{array}{c} \Lambda \end{array}}{\begin{array}{c} \upsilon \end{array}}\\ & -\frac{\begin{array}{c} \mu \end{array}}{\begin{array}{c} \upsilon \end{array}}\left(N\left(t_{j-1}\right)-I\left(t_{j-1}\right)-R\left(t_{j-1}\right)\right)\\ \overset{˘}{S}(t_{j-1})= & N\left(t_{j-1}\right)-\overset{˘}{E}\left(t_{j-1}\right)-I\left(t_{j-1}\right)-R\left(t_{j-1}\right). \end{array} \end{array}$$ (11)

We have the estimators of *E*(*t*_*j*_) and *S*(*t*_*j*_) are given respectively by $\overset{˘}{E}\left(t_{j}\right)$ and $\overset{˘}{S}\left(t_{j}\right)$. For observing that the estimators let fit the data to the model we simulate data based on the SEIR model:

1. Using the function ode of the library deSolve in R we generate the solution from 1 to 100 for the parameters Λ = 4, *β* = 0.2, *υ* = 0.1, *γ* = 0.3 and *μ* = 0.2 and initial value (4, 0, 0.1, 0). These values we note them as *S*(*t*_*i*_), *E*(*t*_*i*_), *R*(*t*_*i*_) with *t*_0_ = 0, …, *t*_100_ = 100
2. Adding 0.2 to *I*(*t*_*i*_) and *R*(*t*_*i*_) with *i* = 1, 3, …, 99. Subtracting 0.2 to *I*(*t*_*i*_) and *R*(*t*_*i*_) with *i* = 0, 2, …, 100.

**Fig 8:**
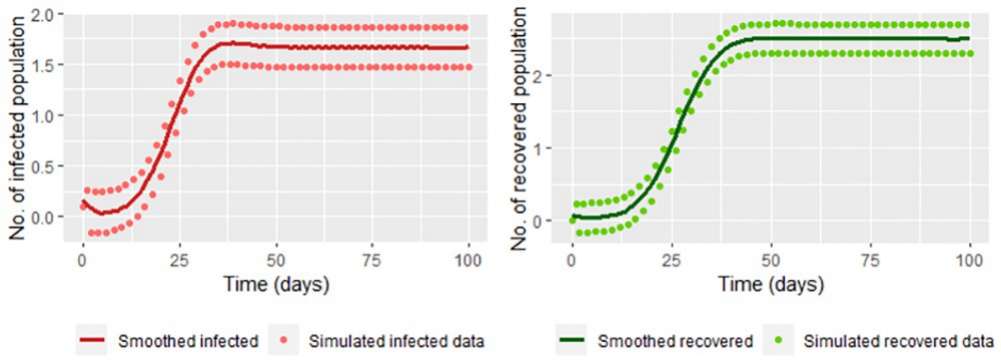
Infected and recovered simulated date built in the steps 1 and 2 and their smoothing.

In the Fig 8 we graph the simulated data in the steps 1 and 2 join with the smoothed functions using frfast in R.

**Fig 9:**
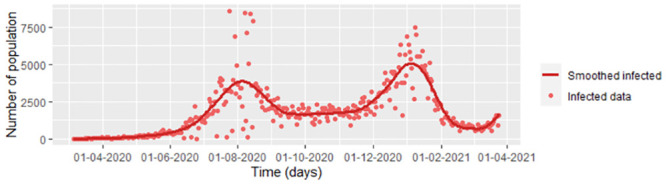
Susceptible and exposed population estimated by $\overset{˘}{S}(t_{j})$ and $\overset{˘}{E}(t_{j})$ (Eq 11) without smoothing infected and recovered data versus solutions for susceptible and recovered of the step 1 (modeled exposed and recovered, respectively).

Now, we calculate $\overset{˘}{S}(t_{j})$ and $\overset{˘}{E}(t_{j})$ (Eq 11) with *t*_*j*_ = 1, …, 100 for comparing the estimation with the “real” data. Fig 9 shows that comparison.

**Fig 10:**
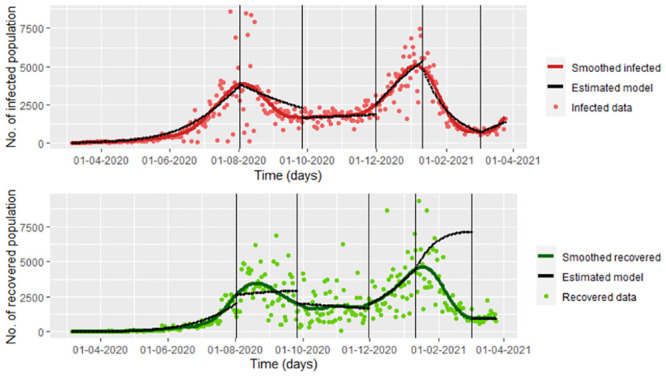
Susceptible and exposed population estimated by $\overset{˘}{S}(t_{j})$ and $\overset{˘}{E}(t_{j})$ (Eq 11) versus solutions for susceptible and recovered of the step 1 (modeled exposed and recovered, respectively).

The susceptible and exposed population is over or underestimated when taking the data directly. We use smoothing techniques to reduce the effect of variations for the infected and recovered data (for example, we use frfast in R) as it can be seen in the Fig 10

Thus, we suggest smoothing the data to estimate the susceptible and exposed population better. On the other hand, we also can have a good estimation by smoothing the values of $\overset{˘}{S}(t_{j})$ and $\overset{˘}{E}(t_{j})$. Note in the Eq 11 we have assumed *υ* as the known parameter. Therefore we would have to estimate *β* and *γ* using the data. We consider the data given by *I*(*t*_*j*_), *R*(*t*_*j*_) and $S(t_{j})\approx \overset{˘}{S}(t_{j})$, $E(t_{j})\approx \overset{˘}{E}(t_{j})$. Thus we can apply the least-squares method by minimizing the equation

$$\begin{array}{c} U=\sum_{i=0}^{n}({\left(\overset{˘}{S}\left(t_{i}\right)-\hat{S}\left(t_{i}\right)\right)}^{2}+{\left(\overset{˘}{E}\left(t_{i}\right)-\hat{E}\left(t_{i}\right)\right)}^{2}+{\left(I\left(t_{i}\right)-\hat{I}\left(t_{i}\right)\right)}^{2}+{\left(R\left(t_{i}\right)-\hat{R}\left(t_{i}\right)\right)}^{2})\text{,}\; \end{array}$$ (12)

taking taking △*t* = *t*_*j*_ − *t*_*j*−1_ for all *j* = 1, …, *n*., we have that *U* is given by

$$\begin{array}{c} \begin{array}{cc} U= & \sum_{j=0}^{n+1}{\left(\overset{˘}{S}\left(t_{j}\right)-\overset{˘}{S}(t_{j-1})-\Lambda △t+\beta \overset{˘}{S}(t_{j-1})I(t_{j-1})△t+\mu \overset{˘}{S}(t_{j-1})△t\right)}^{2}+\\ & \sum_{j=0}^{n+1}{\left(\overset{˘}{E}\left(t_{j}\right)-\overset{˘}{E}(t_{j-1})-\beta \overset{˘}{S}(t_{j-1})I(t_{j-1})△t+\upsilon \overset{˘}{E}(t_{j-1})△t+\mu \overset{˘}{E}(t_{j-1})△t\right)}^{2}+\\ & \sum_{j=1}^{n+1}{\left(I\left(t_{j}\right)-I(t_{j-1})-\upsilon \overset{˘}{E}(t_{j-1})△t+(\mu +\gamma )I(t_{j-1})△t\right)}^{2}+\\ & \sum_{j=1}^{n+1}{\left(R\left(t_{j}\right)-R(t_{j-1})-\gamma I(t_{j-1})△t+\mu R(t_{j-1})△t\right)}^{2}. \end{array} \end{array}$$ (13)

We can find the value of $\hat{\beta }$ by minimizing *U* as follows

$$\begin{array}{c} \begin{array}{cc} \frac{\partial U}{\partial \beta }= & 2\sum_{j=1}^{n+1}(\overset{˘}{S}\left(t_{j}\right)-\overset{˘}{S}(t_{j-1})-\Lambda △t+\beta \overset{˘}{S}(t_{j-1})I(t_{j-1})△t+\mu \overset{˘}{S}(t_{j-1})△t)\\ & \cdot \overset{˘}{S}(t_{j-1})I(t_{j-1})△t+2\sum_{j=1}^{n+1}(\overset{˘}{E}\left(t_{j}\right)-\overset{˘}{E}(t_{j-1})-\beta \overset{˘}{S}(t_{j-1})I(t_{j-1})△t\\ & +\upsilon \overset{˘}{E}(t_{j-1})△t+\mu \overset{˘}{E}(t_{j-1})△t)\cdot \left(-\overset{˘}{S}(t_{j-1})I(t_{j-1})△t\right)=0. \end{array} \end{array}$$ (14)

Obtaining that $\hat{\beta }$ is given by

$$\begin{array}{c} \hat{\beta }=\frac{\begin{array}{c} c \end{array}}{\begin{array}{c} 2△t\sum_{j=1}^{n+1}{\overset{˘}{S}}^{2}(t_{j-1})I^{2}(t_{j-1}), \end{array}} \end{array}$$ (15)

where

$$\begin{array}{cc} c= & (1-\mu △t)\sum_{j=1}^{n+1}{\overset{˘}{S}}^{2}\left(t_{j-1}\right)I\left(t_{j-1}\right)-\sum_{j=1}^{n+1}\overset{˘}{S}(t_{j})\overset{˘}{S}\left(t_{j-1}\right)I\left(t_{j-1}\right)\\ & +\Lambda △t\sum_{j=1}^{n+1}\overset{˘}{S}\left(t_{j-1}\right)I\left(t_{j-1}\right)-(1-\hat{\upsilon }△t-\mu △t)\sum_{j=1}^{n+1}\overset{˘}{E}\left(t_{j-1}\right)\overset{˘}{S}\left(t_{j-1}\right)I\left(t_{j-1}\right)\\ & +\sum_{j=1}^{n+1}\overset{˘}{E}(t_{j})\overset{˘}{S}\left(t_{j-1}\right)I\left(t_{j-1}\right). \end{array}$$

On the other hand, for *γ* we have

$$\begin{array}{c} \begin{array}{cc} \frac{\partial U}{\partial \gamma }= & 2\sum_{j=1}^{n+1}(I\left(t_{j}\right)-I(t_{j-1})-\upsilon E(t_{j-1})△t+(\mu +\gamma )I(t_{j-1})△t)I\left(t_{j-1}\right)△t+\\ & 2\sum_{j=1}^{n+1}(R\left(t_{j}\right)-R(t_{j-1})-\gamma I(t_{j-1})△t+\mu R(t_{j-1})△t)(-I(t_{j-1})△t)=0. \end{array} \end{array}$$ (16)

Obtaining that $\hat{\gamma }$ is given by

$$\begin{array}{c} \hat{\gamma }=\frac{\begin{array}{c} a+\upsilon \sum_{i=1}^{n+1}\overset{˘}{E}(t_{j-1})I\left(t_{j-1}\right) \end{array}}{\begin{array}{c} 2\sum_{i=1}^{n+1}I^{2}(t_{j-1}), \end{array}} \end{array}$$ (17)

where

$$a=\frac{\left(1-\mu △t\right)}{△t}\left(\sum_{i=1}^{n+1}I^{2}\left(t_{j-1}\right)-\sum_{i=1}^{n+1}R\left(t_{j-1}\right)I(t_{j-1})\right)-\frac{1}{△t}\sum_{i=1}^{n+1}I(t_{i})I(t_{j-1})+\frac{1}{△t}\sum_{i=1}^{n+1}R(t_{i})I(t_{j-1}).$$

**Fig 11:**
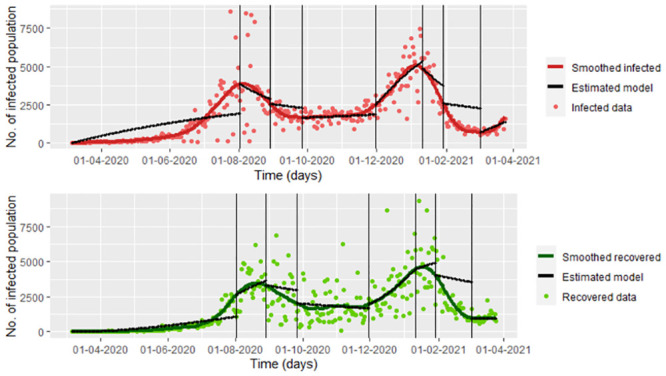
Solution of the system 1 taking Λ = 4, *υ* = 0.1, *μ* = 0.2, $\beta =\hat{\beta }$ and $\gamma =\hat{\gamma }$; where $\hat{\beta }$ and $\hat{\gamma }$ are calculated without smoothing the infected and recovered data simulated (Solution 1). On the other hand, the real solution, which is the solution of the system 1 taking Λ = 4, *υ* = 0.1, *μ* = 0.2, *β* = 0.2 and *γ* = 0.3 (Solution 2) is also graphed.

Note that the estimators are minimum due to the Hessian matrix is given by

$${ℋ}_{\beta ,\gamma }=\left(\begin{array}{cc} 4\sum_{i=1}^{n+1}{\overset{˘}{S}}^{2}(t_{j-1})I^{2}(t_{j-1}){△}^{2}t & 0\\ 0 & 4\sum_{i=1}^{n+1}I^{2}(t_{j-1}){△}^{2}t \end{array}\right)$$

such as $4\sum_{i=1}^{n+1}{\overset{˘}{S}}^{2}(t_{j-1})I^{2}(t_{j-1}){△}^{2}t>0$ and

$$\text{det}({ℋ}_{\beta ,\gamma })=16{△}^{4}t\left(\sum_{i=1}^{n+1}{\overset{˘}{S}}^{2}(t_{j-1})I^{2}(t_{j-1})\right)\left(\sum_{i=1}^{n+1}I^{2}(t_{j-1})\right)>0\text{,}\;$$

therefore $\left(\hat{\beta },\hat{\gamma }\right)$ is a minimum, being these the least squares estimators. In the Fig 11 we graph the solution of the system 1 (using the ode function) taking Λ = 4, *υ* = 0.1, *μ* = 0.2, $\beta \approx \hat{\beta }$ and $\gamma \approx \hat{\gamma }$; where $\hat{\beta }$ and $\hat{\gamma }$ are calculated without smoothing the infected (*I*(*t*_*j*_)) and recovered (*R*(*t*_*j*_)) data simulated in the steps 1 and 2, and whose values are $\hat{\beta }=0.5581449$ and $\hat{\gamma }=0.2645688$. On the same graph, we show the solutions according to ode function with Λ = 4, *υ* = 0.1, *μ* = 0.2, *β* = 0.2 and *γ* = 0.3.

**Fig 12:**
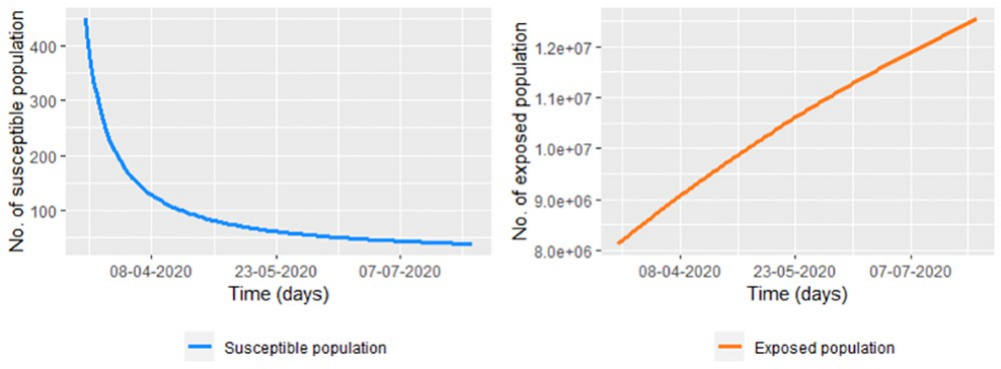
Solution of the system 1 taking Λ = 4, *υ* = 0.1, *μ* = 0.2, $\beta =\hat{\beta }$ and $\gamma =\hat{\gamma }$; where $\hat{\beta }$ and $\hat{\gamma }$ are calculated by smoothing the infected and recovered data simulated (Solution 1). On the other hand, the real solution, which is the solution of the system 1 taking Λ = 4, *υ* = 0.1, *μ* = 0.2, *β* = 0.2 and *γ* = 0.3 (Solution 2) is also graphed.

Note that we don’t have a good estimation for the infected and recovered data without smoothing. It also can be seen that the solution for the susceptible and recovered is far from the corresponding smoothed population estimated. This can be corrected by smoothing the infected and recovered data (in our case, the data simulated by the steps 1 and 2) before calculating $\hat{\beta }$ and $\hat{\gamma }$, whose values are $\hat{\beta }=0.199841$ and $\hat{\gamma }=0.2934448$ close to *β* and *γ*, respectively. Note in Fig 12 that smoothing the data lets a good fit since smoothed data is near to the solution of the system.

**Fig 13:**
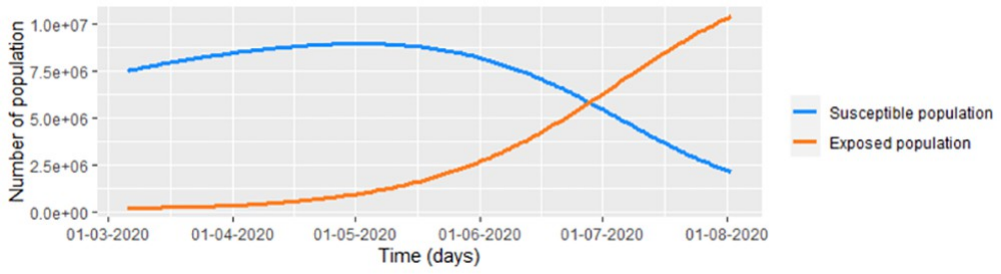
Projected population (according to DANE) from Bogota during the first 385 days of COVID-19 in Bogota. Susceptible and exposed estimations using the Eq 11.

Following the last paragraph, we taking the smoothed infected and recovered data of COVID-19 from Bogota (Fig 2) to estimate the susceptible and exposed population under the pandemic (Fig 13). Calculating $\overset{˘}{S}(t_{j})$ and $\overset{˘}{E}(t_{j})$ (Eq 11) with $\Lambda =(9.42/1000)\bar{p}\approx$ 73,660, *μ* = 4/1000 and the incubation rate of COVID-19 given by *υ* = 1/5.2 (1/*υ* = 5.2 days is the incubation time according to [37]).

Note that the susceptible population always increases. This is why Λ ≫ *μ* max_*i*_(*p*_*i*_) with *p*_*i*_ the projected population from Bogota in the day *i*. The reason why there is such a large increase in the population from Bogota is the migration mostly of people from Venezuela regarding to Migración Colombia [38]. On the other hand, the exposed population presents peaks and valleys which match with the peaks and valleys of the number of infected in Bogota. In the Table 3 we estimate the parameters of the eight intervals under which we consider different infection conditions. Note that the recovered rate could be over-estimated since the values of $\hat{\gamma }$ are more than 1, which indicates that the recuperation time of COVID-19 is less than one day which is false [39].

**Table 3:** Parameter estimation using $\hat{\beta }$ and $\hat{\gamma }$ using the Eqs 15 and 17 for each considered interval *I*_*s*_, *s* = 1, …, 8, respectively. We take $\overset{˘}{S}(t_{j})$ and $\overset{˘}{E}(t_{j})$ according to the Fig 13.

Period of time (*τ*_*s*_)	${\hat{\beta }}_{\tau_{s}}$	${\hat{\gamma }}_{\tau_{s}}$
[1,150)	2.353e-06	8.461e+00
[150,177)	1.600e-06	5.767e+00
[177,205)	2.829e-06	1.019e+01
[205,270)	2.997e-06	1.082e+01
[270,312)	1.309e-06	4.736e+00
[312,330)	1.375e-06	4.981e+00
[330,363)	3.733e-06	1.350e+01
[363,385)	5.105e-06	1.850e+01

Despite using a least-squares method for estimating the parameters and taking some intervals with similar infection conditions, the Table 3 suggests using another estimation method for modeling the COVID-19 with the SEIR model. For this reason, we propose an updated data estimation in the next subsection.

### Parameter estimation on the SEIR model with susceptible, exposed and recovered missing data

In this case, we do not have data corresponding to susceptible, exposed, and recovered populations each time. We could only apply the least squares on the infected population.

$$\begin{array}{c} S=\sum_{j=0}^{n}{\left(I\left(t_{j}\right)-\hat{I}\left(t_{j}\right)\right)}^{2}. \end{array}$$ (18)

However, we would need the values of *E*(*t*_*j*−1_) according to the Eq 8, from where we can see that

$$\begin{array}{c} \begin{array}{cc} \hat{S}(t_{j})+\hat{E}(t_{j})+\hat{R}(t_{j})= & S(t_{j-1})+E(t_{j-1})+R(t_{j-1})+[\Lambda -\mu (S(t_{j-1})+E(t_{j-1})\\ & +R(t_{j-1}))-\upsilon E(t_{j-1})+\gamma I(t_{j-1})](t_{j}-t_{j-1}) \end{array} \end{array}$$ (19)

Replacing *S*(*t*_*j*−1_) + *E*(*t*_*j*−1_) + *R*(*t*_*j*−1_) by *N*(*t*_*j*−1_) − *I*(*t*_*j*−1_) and $\hat{S}\left(t_{j}\right)+\hat{E}\left(t_{j}\right)+\hat{R}\left(t_{j}\right)$ by *N*(*t*_*j*_) − *I*(*t*_*j*_), we obtain an estimator for *E*(*t*_*j*−1_)

$$\begin{array}{c} \begin{array}{cc} \overset{⌣}{E}(t_{j-1})= & \frac{\begin{array}{c} N(t_{j-1})-N(t_{j})-I(t_{j-1})+I(t_{j}) \end{array}}{\begin{array}{c} \upsilon (t_{j}-t_{j-1}) \end{array}}+\frac{\begin{array}{c} \Lambda \end{array}}{\begin{array}{c} \upsilon \end{array}}\\ & -\frac{\begin{array}{c} \mu \end{array}}{\begin{array}{c} \upsilon \end{array}}[N(t_{j-1})-I(t_{j-1})+\gamma I(t_{j-1})] \end{array} \end{array}$$ (20)

Note that we assumed *γ* and *υ* as known parameters. In such manner, we can estimate *R*(*t*_*j*−1_) by $\hat{R}\left(t_{j-1}\right)$ (Eq 8) taking *R*(*t*_0_) = 0 and *S*(*t*_*j*−1_) by $\overset{⌣}{S}(t_{j-1})=N(t_{j-1})-\overset{⌣}{E}\left(t_{j-1}\right)-I\left(t_{j-1}\right)-\hat{R}\left(t_{j-1}\right)$. We want to minimize for finding an estimator for *β*

$$\begin{array}{c} V=\sum_{j=0}^{n}({\left(\overset{⌣}{S}\left(t_{j}\right)-\hat{S}\left(t_{j}\right)\right)}^{2}+{\left(\overset{⌣}{E}\left(t_{j}\right)-\hat{E}\left(t_{j}\right)\right)}^{2}+{\left(I\left(t_{j}\right)-\hat{I}\left(t_{j}\right)\right)}^{2}+{\left(R\left(t_{j}\right)-\hat{R}\left(t_{j}\right)\right)}^{2})\text{,}\; \end{array}$$ (21)

where $\overset{˘}{R}(t_{i})=\hat{R}(t_{i})$. Actually, independently if we have $\overset{˘}{R}(t_{i})\neq \hat{R}(t_{i})$, for minimizing *V* we have that the derivative respect to *β* of $\sum_{i=0}^{n}{\left(\hat{R}(t_{i})-\hat{R}(t_{i})\right)}^{2}$ is equal to 0 since *β* does not appear at $\hat{R}(t_{i})$.

Analogously, following what we did for minimizing *U* on the Eq 12 we have that $\hat{\beta }$ is given by the Eq 15, which is a minimum due to $\partial^{2}V/\partial \beta^{2}=4\sum_{i=1}^{n+1}{\overset{⌣}{S}}^{2}(t_{j-1})I^{2}(t_{j-1})>0$.

**Fig 14:**
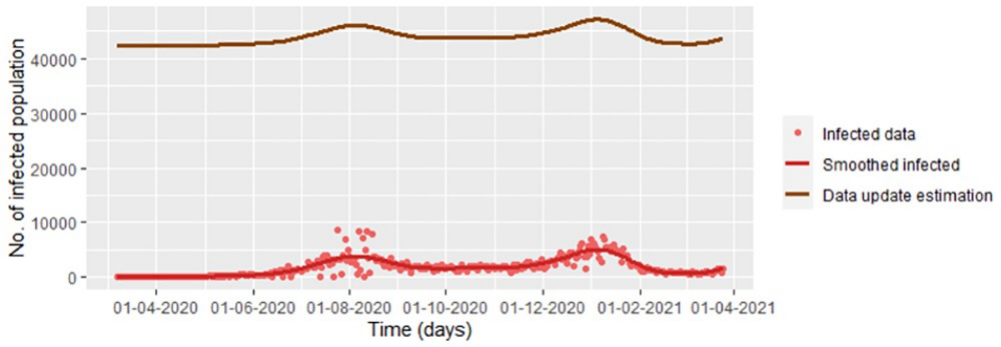
Solution of the system 1 taking Λ = 4, *υ* = 0.1, *μ* = 0.2, *γ* = 0.3 and $\beta =\hat{\beta }$ (Solution 1). $\hat{\beta }$ is calculated using $\overset{⌣}{S}(t_{j})$, $\overset{⌣}{E}(t_{j})$ (based on the smoothed *I*(*t*_*j*_)) and the infected data without smoothing. On the other hand, the real solution, which is the solution of the system 1 taking Λ = 4, *υ* = 0.1, *μ* = 0.2, *β* = 0.2 and *γ* = 0.3 (Solution 2) is also graphed.

In Fig 14, we graph the estimations for the susceptible ($\overset{⌣}{S}(t_{j})$), exposed ($\overset{⌣}{E}(t_{j})$) and recovered ($\hat{R}(t_{j})$) population, from the infected data simulated in the steps 1 and 2 (considering unknown recovered data). In addition, we graph the solution of the system taking the parameters as the step 1, except for *β*, which is taken by $\beta \approx \hat{\beta }=0.1397632$ calculated of the Eq 15, under $\overset{˘}{S}(t_{j})\approx \overset{⌣}{S}(t_{j})$, $\overset{˘}{E}(t_{j})\approx \overset{⌣}{E}(t_{j})$ and the infected data without smoothing.

**Fig 15:**
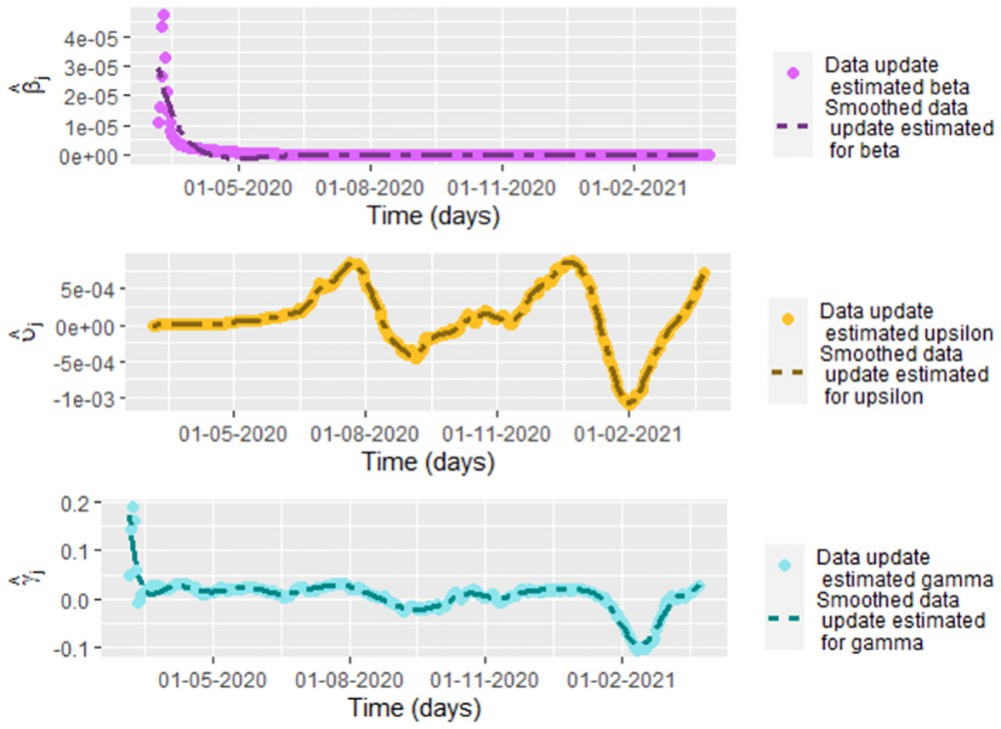
Solution of the system 1 taking Λ = 4, *υ* = 0.1, *μ* = 0.2, *γ* = 0.3 and $\beta =\hat{\beta }$ (Solution 1). $\hat{\beta }$ is calculated using $\overset{⌣}{S}(t_{j})$, $\overset{⌣}{E}(t_{j})$ (based on the smoothed *I*(*t*_*j*_)) and the smoothed infected data. On the other hand, the real solution, which is the solution of the system 1 taking Λ = 4, *υ* = 0.1, *μ* = 0.2, *β* = 0.2 and *γ* = 0.3 (Solution 2) is also graphed.

If we take the estimations for the susceptible ($\overset{⌣}{S}(t_{j})$), exposed ($\overset{⌣}{E}(t_{j})$) and recovered ($\hat{R}(t_{j})$) population, calculated only from the smoothed infected data (from the infected data simulated in the steps 1 and 2) using the function frfast from the package npregfast in R. We also calculate $\hat{\beta }$ using $\overset{˘}{S}(t_{j})\approx \overset{⌣}{S}(t_{j})$, $\overset{˘}{E}(t_{j})\approx \overset{⌣}{E}(t_{j})$ (using the smoothed simulated data) and the smoothed infected data, and so $\beta \approx \hat{\beta }=0.1397632$, which is too far from *β* = 0.2. That implies that not necessarily smooth the data drives to a good fit (see Fig 15).

Note in the bottom right of the Fig 15 that there is a good estimation for the recovered population, being too close $\hat{R}(t_{j})$ to *R*(*t*_*j*_). We can estimate the susceptible and exposed population using the Eq 22.

$$\begin{array}{c} \begin{array}{cc} \overset{↼}{E}(t_{j-1})= & \frac{\begin{array}{c} N\left(t_{j-1}\right)-N\left(t_{j}\right)-I\left(t_{j-1}\right)+I\left(t_{j}\right)-\hat{R}\left(t_{j-1}\right)+\hat{R}\left(t_{j}\right) \end{array}}{\begin{array}{c} \upsilon (t_{j}-t_{j-1}) \end{array}}+\frac{\begin{array}{c} \Lambda \end{array}}{\begin{array}{c} \upsilon \end{array}}\\ & -\frac{\begin{array}{c} \mu \end{array}}{\begin{array}{c} \upsilon \end{array}}\left(N\left(t_{j-1}\right)-I\left(t_{j-1}\right)-\hat{R}\left(t_{j-1}\right)\right)\\ \overset{↼}{S}(t_{j-1})= & N\left(t_{j-1}\right)-\overset{↼}{E}\left(t_{j-1}\right)-I\left(t_{j-1}\right)-\hat{R}\left(t_{j-1}\right). \end{array} \end{array}$$ (22)

**Fig 16:**
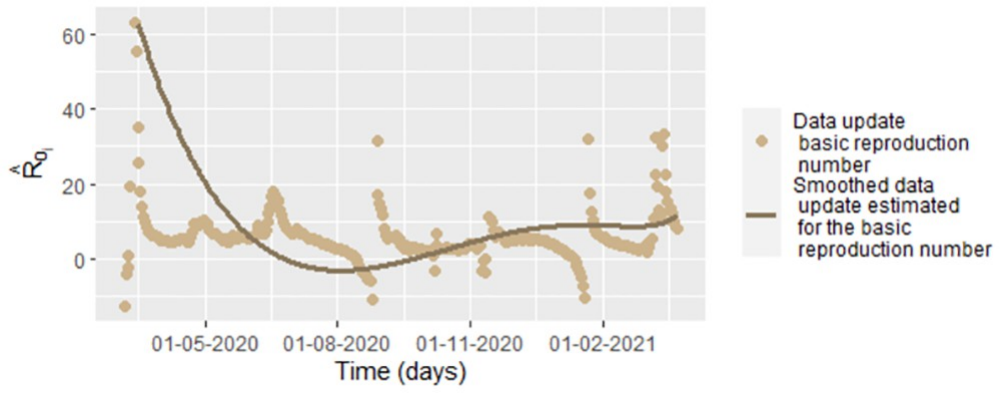
Solution of the system 1 taking Λ = 4, *υ* = 0.1, *μ* = 0.2, *γ* = 0.3 and $\beta =\hat{\beta }$ (Solution 1). $\hat{\beta }$ is calculated using $\overset{↼}{S}\left(t_{j}\right)$, $\overset{↼}{E}\left(t_{j}\right)$ (based on the smoothed *I*(*t*_*j*_)) and the smoothed infected data. On the other hand, the real solution, which is the solution of the system 1 taking Λ = 4, *υ* = 0.1, *μ* = 0.2, *β* = 0.2 and *γ* = 0.3 (Solution 2) is also graphed.

Estimating the susceptible, exposed and recovered population respectively by $\overset{↼}{S}\left(t_{j}\right)$, $\overset{↼}{R}\left(t_{j}\right)$ and $\hat{R}\left(t_{j}\right)$, note in the Fig 16 that these populations are being good estimated. Calculating $\hat{\beta }$ (Eq 15) based on $\overset{↼}{S}\left(t_{j}\right)$, $\overset{↼}{R}\left(t_{j}\right)$ and the smoothed infected data, we have $\hat{\beta }=0.2002431$, which is close to *β* = 0.2 which with the Fig 16 indicate a good estimation.

### A data update approach

Exogenous variables such as vaccination and quarantines interact with the infections, and we did not consider them in the present model. However, the SEIR model is one of the simplest to describe an epidemic’s behavior. For this reason, we propose a data update method of parameter estimation based on previous approximations to the unknown populations who are susceptible and exposed. This method estimates the parameters for each time *t*_*j*_, based on the available data of the previous day. Its implementation is different for each model, and it depends on the previous known parameters. This focus was approached by [40], under a regression Poisson model applied only on age groups, to predict the number of cases and deaths of COVID-19 in Italy, taking as regression variable the time. [41] estimate and map the prevalence of Chagas disease among adults in the United States, based on some small population subgroups at the public micro-area (PUMA) level for mapping. [42] uses Markov Chain Monte Carlo (MCMC) method to fit an SEIR-type model to the data of the cumulative number of laboratory-confirmed 2019-nCov cases from the National Health Commission of the People’s Republic of China. Finally, [43] suggests using a bayesian model based on Newtonian equations of an ordinary differential system to estimate the parameters. In this paper, we have given a general estimation of the parameters. However, it is not used with real data. In our paper, we propose a novel method whose purpose is estimating each parameter on time *t*_*j*_ from infected and recovered data of the previous day, that is *I*(*t*_*j*−1_) and *R*(*t*_*j*−1_). We are going to consider $\hat{S}(t_{j})\approx \overset{˘}{S}(t_{j})$, $\hat{E}(t_{j})\approx \overset{˘}{E}(t_{j})$, $\hat{I}(t_{j})\approx I(t_{j})$ and $\hat{R}(t_{j})\approx R(t_{j})$ to estimate the parameters based on a data update for *β* and *γ*. Solving for *γ* on the Eq 8 we have

$$\begin{array}{c} {\hat{\gamma }}_{j}=\frac{R\left(t_{j}\right)-R\left(t_{j-1}\right)+\mu R\left(t_{j-1}\right)△t}{I(t_{j-1})△t}\text{.} \end{array}$$ (23)

In the Eq 23, we fit the recovered data using the data update approach. On the other hand, we solve *β* from the Eq 8 as follows

$$\begin{array}{c} {\tilde{\beta }}_{j}=\frac{\overset{˘}{S}\left(t_{j-1}\right)-\overset{˘}{S}\left(t_{j}\right)+\Lambda △t-\mu \overset{˘}{S}\left(t_{j-1}\right)△t}{\overset{˘}{S}\left(t_{j-1}\right)I\left(t_{j-1}\right)△t}\text{.} \end{array}$$ (24)

**Fig 17:**
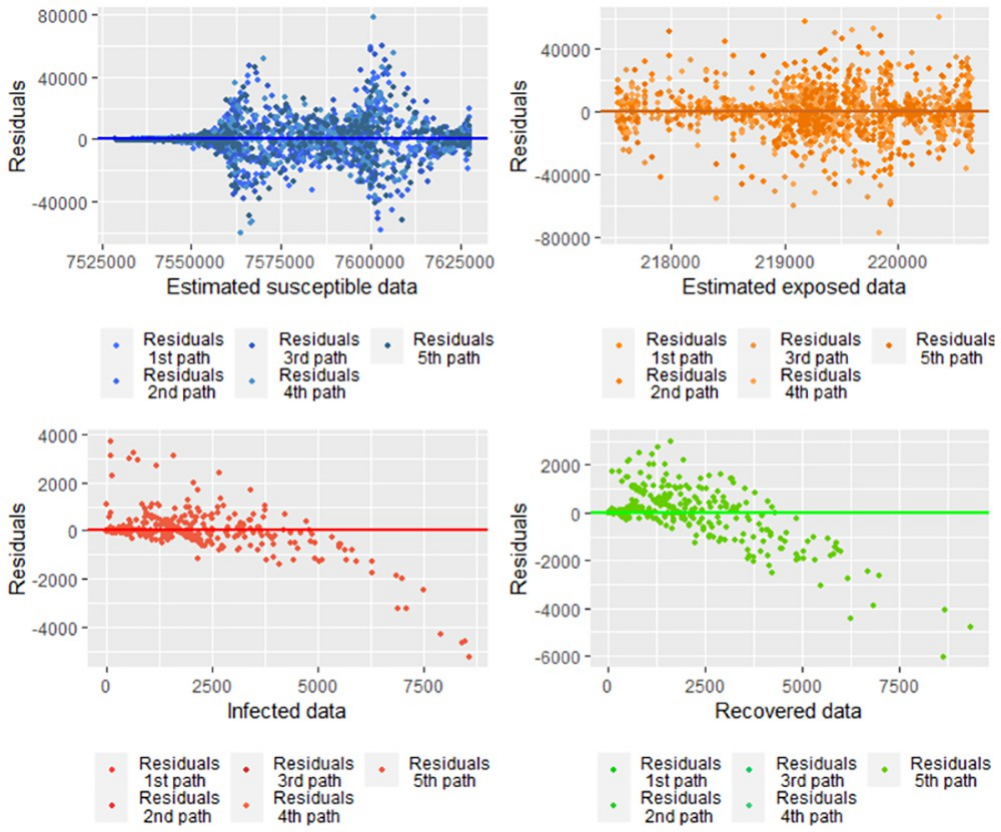
Estimated infected population under the Eq 25 for smoothed COVID-19 Bogota data using the estimators given by *υ* = 1/5.2, ${\hat{\beta }}_{j}$ and ${\hat{\gamma }}_{j}$ 24 and 23, respectively.

In the Eq 24, we fit the susceptible estimated data using the data update approach. Eq 25 is based completely in the Eq 8, which was obtained of replacing *β* and *γ* by ${\tilde{\beta }}_{j}$ and ${\hat{\gamma }}_{j}$, respectively. In Fig 17, we graph the values given by 25 with the infected data and its smoothing.

$$\begin{array}{c} \begin{array}{c} \overset{*}{S}(t_{j})=\overset{˘}{S}(t_{j-1})+[\Lambda -{\tilde{\beta }}_{j}\overset{˘}{S}(t_{j-1})I(t_{j-1})-\mu \overset{˘}{S}(t_{j-1})](t_{j}-t_{j-1})\\ \overset{*}{E}(t_{j})=\overset{˘}{E}(t_{j-1})+[{\tilde{\beta }}_{j}\overset{˘}{S}(t_{j-1})I(t_{j-1})-\upsilon \overset{˘}{E}(t_{j-1})-\mu \overset{˘}{E}(t_{j-1})](t_{j}-t_{j-1})\\ \overset{*}{I}(t_{j})=I(t_{j-1})+[\upsilon \overset{˘}{S}(t_{j-1})-(\mu +{\hat{\gamma }}_{j})I(t_{j-1})](t_{j}-t_{j-1})\\ \overset{*}{R}(t_{j})=R(t_{j-1})+[{\hat{\gamma }}_{j}I(t_{j-1})-\mu R(t_{j-1})](t_{j}-t_{j-1})\text{,} \end{array} \end{array}$$ (25)

However, when we compare with the COVID-19 data for Bogota, we realize that this does not work for the infected population for which there is an overestimation, as can be seen in Fig 17. Reason why there is an over-estimation is that $\overset{˘}{S}(t_{j-1})-\overset{˘}{S}(t_{j})$ is a big number in comparison with $\Lambda △t-\mu \overset{˘}{S}(t_{j-1})$. On the other hand, despite having a good estimation for the susceptible population, it is important to say that there are exogenous variables that were not considered, in particular, the vaccination which started in February 17^th^/2021, reason which the infected population avoids of being good estimated. However, the focus given by the equations 24 and 23 let us estimate when there were peaks and valleys, in spite of the over-estimation of the infected population.

Estimators given by 24 and 23 fit only the susceptible and the recovered population, which is why the infected and the exposed population is not well estimated. Therefore, we propose an estimation, but considering *β*, *υ*, and *γ* under other data update approach, for which *γ* is fitted for the recovered population, *υ* for the infected population and *β* for the exposed population.

It is important to highlight that *υ* is being re-estimated because we have to use the exposed population for the estimation. We could consider the values of $\overset{˘}{E}(t_{j})$ as an indication of the behavior of the exposed population. Solving for *γ*, *υ* and *β* on the Eq 8 we obtain ${\hat{\gamma }}_{j}$, ${\hat{\upsilon }}_{j}$ and ${\hat{\beta }}_{j}$ given respectively by 23,

$$\begin{array}{c} {\hat{\upsilon }}_{j}=\frac{I\left(t_{j}\right)-I\left(t_{j-1}\right)+\left(\mu +{\hat{\gamma }}_{j}\right)I\left(t_{j-1}\right)△t}{\overset{˘}{E}\left(t_{j-1}\right)△t}\text{,}\; \end{array}$$ (26)

and

$$\begin{array}{c} {\hat{\beta }}_{j}=\frac{\overset{˘}{E}\left(t_{j}\right)-\overset{˘}{E}\left(t_{j-1}\right)+\left({\hat{\upsilon }}_{j}+\mu \right)\overset{˘}{E}\left(t_{j-1}\right)△t}{\overset{˘}{S}\left(t_{j-1}\right)I\left(t_{j-1}\right)△t}\text{.} \end{array}$$ (27)

**Fig 18:**
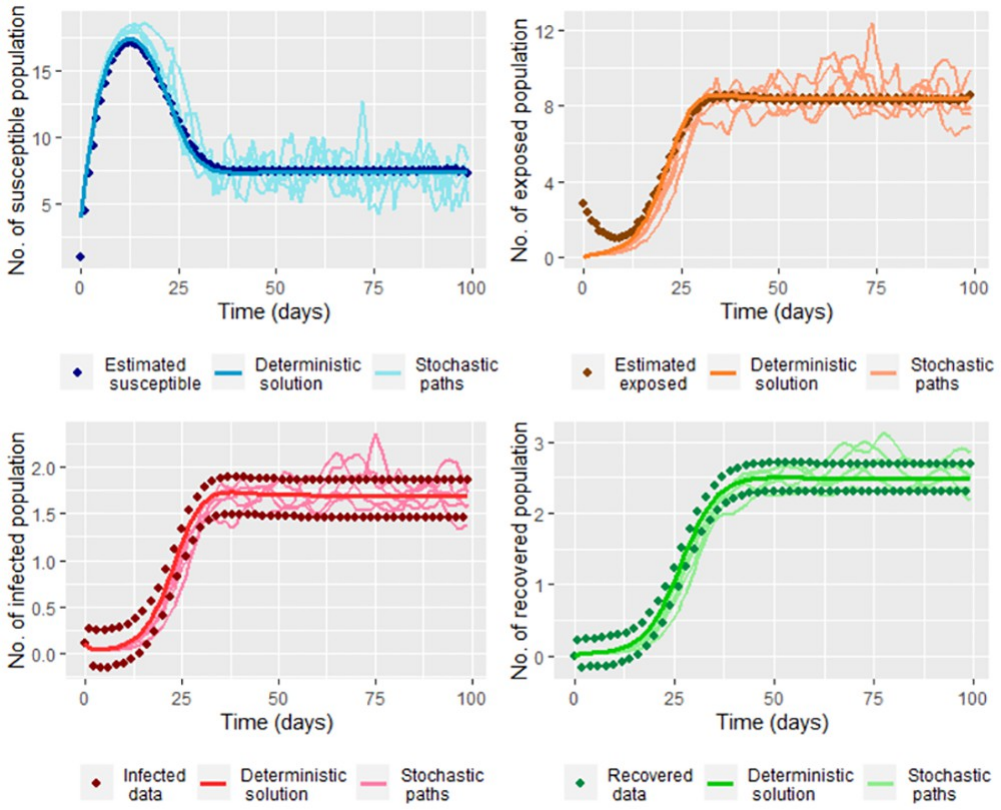
Estimated populations under the Model 3 for smoothed COVID-19 Bogota data using the estimators given by ${\hat{\beta }}_{j}$, ${\hat{\upsilon }}_{j}$ and ${\hat{\gamma }}_{j}$ given by 27, 26 and 23, respectively.

For smoothed infected and recovered COVID-19 data from Bogota note that there is a good fitting between the estimation by the data update approach method and the data, as you can see in the Fig 18. We graph the data update estimation using the Eq 28 which is a version of 8 replacing *β*, *υ*, *γ*, *S*(*t*_*j*_) and *E*(*t*_*j*_) by ${\hat{\beta }}_{j}$, ${\hat{\upsilon }}_{j}$, ${\hat{\gamma }}_{j}$ (from the Eqs 23, 26 and 27, respectively), $\overset{˘}{S}(t_{j})$ and $\overset{˘}{E}(t_{j})$ (from the Eq 11).

$$\begin{array}{c} \begin{array}{c} \text{Model}\;\text{3:}\\ \overset{⌢}{S}(t_{j})=\overset{˘}{S}(t_{j-1})+[\Lambda -{\hat{\beta }}_{j}\overset{˘}{S}(t_{j-1})I(t_{j-1})-\mu \overset{˘}{S}(t_{j-1})](t_{j}-t_{j-1})\\ \overset{⌢}{E}(t_{j})=\overset{˘}{E}(t_{j-1})+[{\hat{\beta }}_{j}\overset{˘}{S}(t_{j-1})I(t_{j-1})-{\hat{\upsilon }}_{j}\overset{˘}{E}(t_{j-1})-\mu \overset{˘}{E}(t_{j-1})](t_{j}-t_{j-1})\\ \overset{⌢}{I}(t_{j})=I(t_{j-1})+[{\hat{\upsilon }}_{j}\overset{˘}{S}(t_{j-1})-(\mu +{\hat{\gamma }}_{j})I(t_{j-1})](t_{j}-t_{j-1})\\ \overset{⌢}{R}(t_{j})=R(t_{j-1})+[{\hat{\gamma }}_{j}I(t_{j-1})-\mu R(t_{j-1})](t_{j}-t_{j-1})\text{.} \end{array} \end{array}$$ (28)

Note that the model 3 given by the system 28 has a perfect fit for the indication of the exposed population. Thus, if we had the exposed data, we would have a good fit by smoothing the data.

**Fig 19:**
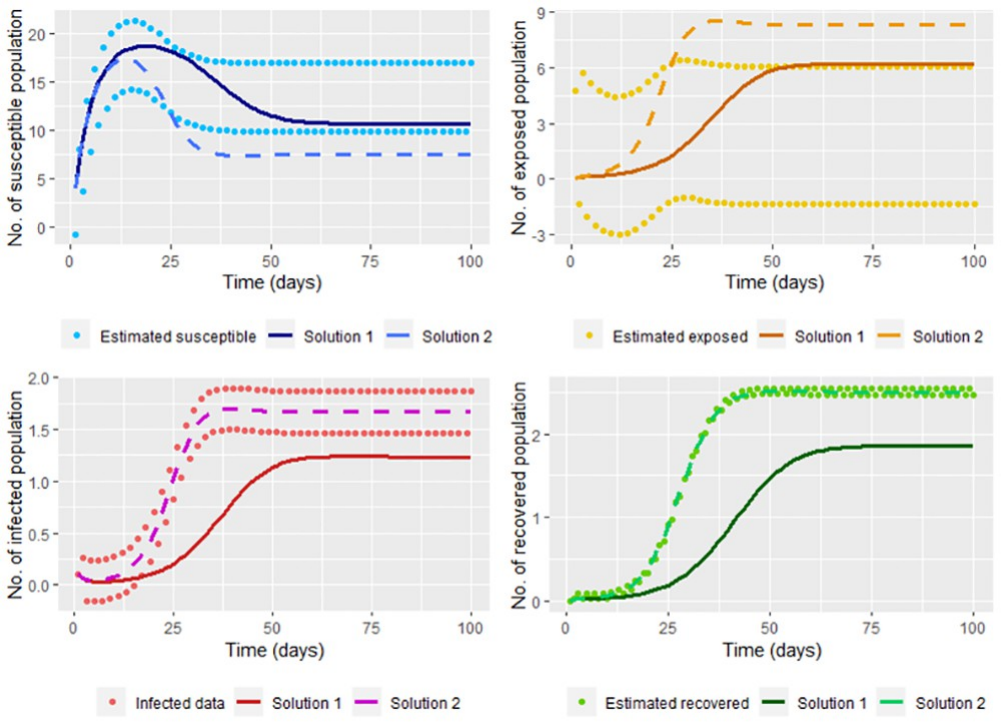
A update data susceptible estimation based on $N(t_{j})-\overset{ˇ}{E}(t_{j})-\overset{ˇ}{I}(t_{j})-\overset{ˇ}{R}(t_{j})$ with $\overset{ˇ}{E}(t_{j})$, $\overset{ˇ}{I}(t_{j})$ and $\overset{ˇ}{R}(t_{j})$ given by the update data method of estimation for *E*(*t*_*j*_), *I*(*t*_*j*_) and *R*(*t*_*j*_), respectively.

On the other hand, the susceptible population is being over-estimated on average by 42 084 of the population. That is because we did the data update for the indication of the exposed population but not for the indication of the susceptible population. For improving the susceptible estimation we use that $S(t_{j})\approx N(t_{j})-\overset{ˇ}{E}(t_{j})-\overset{ˇ}{I}(t_{j})-\overset{ˇ}{R}(t_{j})$ with $\overset{ˇ}{E}(t_{j})$, $\overset{ˇ}{I}(t_{j})$ and $\overset{ˇ}{R}(t_{j})$ are the update data estimation of *E*(*t*_*j*_), *I*(*t*_*j*_) and *R*(*t*_*j*_), respectively. In the Fig 19 we see that there is a good fit between the indication susceptible data and the values of $N(t_{j})-\overset{ˇ}{E}(t_{j})-\overset{ˇ}{I}(t_{j})-\overset{ˇ}{R}(t_{j})$. Note in the Fig 19 that the susceptible population is predicted to always increase. This due to we do not consider vaccination or other measures to control the epidemic.

For each one of the eight intervals that we take with different infection conditions of COVID-19 (Fig 6), we calculate the mean and the standard deviation for each parameter. Note in the Table 4 the means of the incubation and the recuperation rates are negative when as the number of infected as of recovered population decrease, that is, on the intervals [177, 205), [312, 330) and [330, 363). For the rest of the intervals, all the parameters are positive, even when only the infected population decreases as happens on the interval [150, 177) (see Fig 6).

**Table 4:** Mean and standard deviation of the values of ${\hat{\beta }}_{j}$, ${\hat{\upsilon }}_{j}$ and ${\hat{\gamma }}_{j}$ given by 27, 26 and 23, respectively, for each period of time of the Fig 6.

$\begin{array}{c} \text{Period}\\ \left(\tau_{s}\right) \end{array}$	Mean of ${\hat{\beta }}_{j}$	SD of ${\hat{\beta }}_{j}$	Mean of ${\hat{\upsilon }}_{j}$	SD of ${\hat{\upsilon }}_{j}$	Mean of $\hat{\gamma_{j}}$	SD of ${\hat{\gamma }}_{j}$
[1,150)	2.382e-06	6.666e-06	2.208e-04	2.756e-04	2.313e-02	2.222e-02
[150,177)	3.276e-08	3.003e-09	4.669e-06	2.996e-04	9.426e-03	9.603e-03
[177,205)	5.816e-08	9.896e-09	-2.669e-04	1.233e-04	-1.859e-02	4.833e-03
[205,270)	6.644e-08	4.498e-09	1.230e-04	1.539e-04	2.942e-03	1.131e-02
[270,312)	3.500e-08	1.002e-08	6.441e-04	2.076e-04	1.770e-02	1.749e-03
[312,330)	2.382e-08	3.285e-09	-5.584e-04	3.532e-04	-5.225e-03	1.503e-02
[330,363)	1.062e-07	4.868e-08	-6.097e-04	3.689e-04	-7.177e-02	2.921e-02
[363,385)	1.395e-07	3.025e-08	2.774e-04	2.279e-04	7.101e-03	8.571e-03

**Fig 20:**
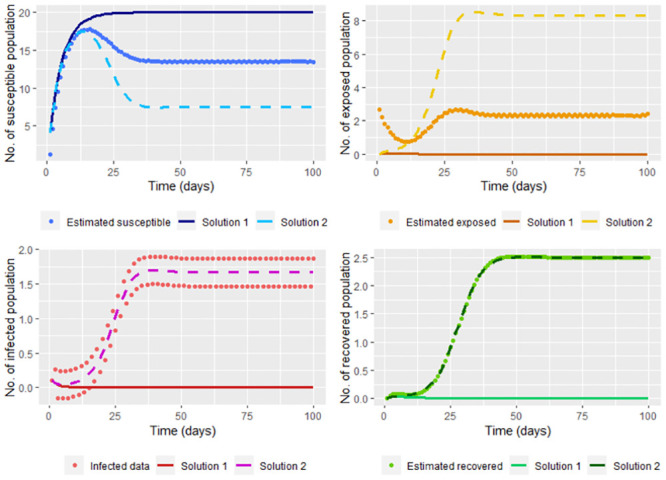
Graphs of ${\hat{\beta }}_{j}$, ${\hat{\upsilon }}_{j}$ and ${\hat{\gamma }}_{j}$ given by 27, 26 and 23, respectively, with respect to the time for smoothed COVID-19 data from Bogota.

In some papers as [27–31] are considered the parameters as functions depending on the time. We can think *β*, *υ* and *γ* as functions, whose graphs for COVID-19 data from Bogota is showed in the Fig 20. We also could interpret *β*, *υ* and *γ* under the focus of time series, estimating the corresponding parameters using the data given by *β*_*j*_, *υ*_*j*_ and *γ*_*j*_ calculated respectively by 27, 26 and 23. In the Fig 20, we also graph the smoothed functions of *β*_*j*_, *υ*_*j*_ and *γ*_*j*_ using the function frfast in R.

**Fig 21:**
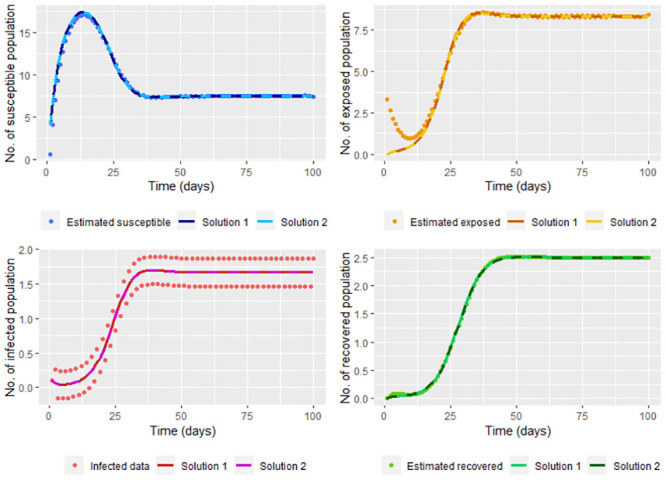
Graphs of the estimated basic reproduction number, ${\hat{R}}_{0_{j}}=\frac{{\hat{\upsilon }}_{j}{\hat{\beta }}_{j}\Lambda }{\mu ({\hat{\upsilon }}_{j}+\mu )({\hat{\gamma }}_{j}+\mu )}$, with ${\hat{\beta }}_{j}$, ${\hat{\upsilon }}_{j}$ and ${\hat{\gamma }}_{j}$ given by 27, 26 and 23, respectively. ${\hat{R}}_{0_{j}}$ is graphed with respect to the time for smoothed COVID-19 data from Bogota.

Under the values of *β*_*j*_, *υ*_*j*_ and *γ*_*j*_ it is not possible to model *β*, *υ* and *γ* as random variables since the *β*_*j*_, *υ*_*j*_ and *γ*_*j*_ are not independent since the parameters depend on the number on the infected and recovered people on the previous days. In the Fig 21 we graph the basic reproduction number by data update estimation, ${\hat{R}}_{0_{j}}=\frac{{\hat{\upsilon }}_{j}{\hat{\beta }}_{j}\Lambda }{\mu ({\hat{\upsilon }}_{j}+\mu )({\hat{\gamma }}_{j}+\mu )}$, (see [44]) for each day, where ${\hat{\beta }}_{j}$, ${\hat{\upsilon }}_{j}$ and ${\hat{\gamma }}_{j}$ given by 27, 26 and 23, respectively. In the same figure we also show the corresponding smoothing using the function frfast in R. Similarly that for *β*, *υ* and *γ* estimate by data update, we also we think under this focus that *R*_0_ is a time series or a function depending on the time, but not a random variable.

In the Table 5 we calculate the mean and the standard deviation of the data update estimation of the basic reproduction number and for the corresponding smoothing. Interpreting the basic reproduction number ([45]), we conclude that the COVID-19 has been contagious high in Bogota, even when the number of infected population has decreased. The conditions for fewer infections are when the number of infected populations decreases and the recovered increases (interval [150, 177)).

- *“If, R*_0_ < 1 *then on average an infected individual produces less than one new infected individual throughout its infectious period, and the infection cannot grow”* [45],
- *“If R*_0_ > 1 *then each infected individual produces, on average, more than one new infection, and the disease can invade the population”* [45],

**Table 5:** Mean and standard deviation of update data estimations of the basic reproduction number, ${\hat{R}}_{0_{j}}=\frac{{\hat{\upsilon }}_{j}{\hat{\beta }}_{j}\Lambda }{\mu ({\hat{\upsilon }}_{j}+\mu )({\hat{\gamma }}_{j}+\mu )}$ (with ${\hat{\beta }}_{j}$, ${\hat{\upsilon }}_{j}$ and ${\hat{\gamma }}_{j}$ given by 27, 26 and 23, respectively) for smoothed COVID-19 data from Bogota in the eight intervals considered in the Fig 6.

$\begin{array}{c} \text{Period}\\ \left(\tau_{s}\right) \end{array}$	Mean of ${\hat{R}}_{0_{j}}$	SD of ${\hat{R}}_{0_{j}}$	$\begin{array}{c} \text{Mean of}\\ \text{smoothed}\;{\hat{R}}_{0_{j}} \end{array}$	$\begin{array}{c} \text{SD of}\\ \text{smoothed}\;{\hat{R}}_{0_{j}} \end{array}$
[1,150)	18.353	150.219	19.318	22.827
[150,177)	1.267	23.723	-2.726	0.299
[177,205)	5.834	3.851	-0.942	0.764
[205,270)	5.122	13.458	3.879	2.014
[270,312)	4.133	1.591	8.249	0.587
[312,330)	1.417	13.168	9.019	0.017
[330,363)	5.848	10.689	8.806	0.097
[363,385)	16.710	13.879	9.761	0.904

For interpreting the basic reproduction number, we can establish that the COVID-19 has invaded the population, and produced a state of endemicity in Bogota. If the infection conditions continue as in the period [363, 385), the epidemic could invade the population; unless control and mitigation measures like vaccination and isolation are applied. Actually, in Bogota, 55.2% of the population has been vaccinated according to Secretaría de Salud de Bogota data [46], what it would hope that *R*_0_ < 1, if it is calculated for the data from august 24^th^/2021 to October 13^rd^/2021. On the other hand, in Table 5 can be seen that there is significant variability of ${\hat{R}}_{0}$ in particular for the first period, which means that the infection conditions can change considerably between one day and another.

## SEIR model with random perturbations and its estimation

The above SIR and SEIR models are *deterministic*. However, epidemics tend to occur in cycles of outbreaks due to variations in the infection rate mainly related to certain external factors, such as people’s social activities and climatic fluctuations. The *climatic variations* can affect the *infection rate* *β*. According to [47], “many pathogens causing needle diseases are sensitive to precipitation and humidity, and their rates of reproduction, spread, and infection are greater when conditions are moist”. More recently reported, including media about the evidence of the mechanism by which climate change could have played a direct role in the emergence of SARs-CoV-2 [48–52]. Actually, [53] suggests that the climatology parameters could potentially affect the spread of the COVID-19. On the other hand, in [16] is highlight that the deterministic models “*do not involve the variability of the sources of the information nor the possible errors and biases*”, therefore, we model also the infection rate randomly. Some studies as [54–56] use the Brownian motion to model spatial-temporally as the temperature and weather variations affect the pollen dynamic, and the infection rate on epidemic; using equations similarly to 29 to some model parameters. This was applied even on partial differential systems. We consider the infection rate as a stochastic parameter (through random perturbations) and equation is given by

$$\begin{array}{c} \tilde{\beta }:=\beta +\sigma B(t), \end{array}$$ (29)

Where *β* and *σ* are positive constants, and {*B*(*t*)}_*t*≥0_ is the *standard Brownian motion* on the probability space (Ω,ℑ,ℙ,) which is driving the fluctuations in the dynamics of the epidemic. As we know that *dB*(*t*) is the increment of the standard Brownian motion and is normally distributed. Also, the parameter *β* is the rate of transmission of infection, and *σ* is the *volatility parameter* which describes the amount of uncertainty of the parameter *β*. Now, we replace *βdt* by *βdt* + *σdB*(*t*) in the system 1, we now propose the following system of stochastic differential equations for the *SEIR model with random perturbations*, whose system is the Eq 30.

$$\begin{array}{c} \begin{array}{l} dS(t)=\left(\Lambda -\beta I(t)S(t)-\mu S\left(t\right)\right)dt-\sigma I(t)S(t)dB\left(t\right)\\ dE(t)=\left(\beta I(t)S(t)-\upsilon E\left(t\right)-\mu E\left(t\right)\right)dt+\sigma I(t)S(t)dB\left(t\right)\\ dI(t)=\left(\upsilon E\left(t\right)-\gamma I(t)-\mu I\left(t\right)\right)dt\\ dR(t)=\left(\gamma I(t)-\mu R\left(t\right)\right)dt, \end{array} \end{array}$$ (30)

Our model considers environmental variations and social behaviors in the infection rate, inside of a Brownian motion with a volatility parameter. In [57] such variations are modeled by the Eq 29. One weakness of modeling infection rate by using random perturbations is that to big values of volatility parameter, it will have big values for *E*(*t*) or *I*(*t*), therefore we expect small values when we implement it to real data. In the next subsection 1, an estimator of *σ* by minimizing the sum of ordinary squares and using the estimators of the susceptible and exposed population given by 11.

### Estimation of volatility parameter

We can approximate the populations under the SEIR model with random perturbations by using the approximations given by the Euler-Maruyama method [36]:

$$\begin{array}{cc} \tilde{S}(t_{j})= & S(t_{j-1})+[\Lambda -\beta S(t_{j-1})I(t_{j-1})-\mu S(t_{j-1})](t_{j}-t_{j-1})-\sigma S(t_{j-1})I(t_{j-1})\\ & \cdot (B(t_{j})-B(t_{j-1}))=\hat{S}(t_{j})-\sigma S(t_{j-1})I(t_{j-1})(B(t_{j})-B(t_{j-1}))\\ \tilde{E}(t_{j})= & E(t_{j-1})+[\beta S(t_{j-1})I(t_{j-1})-(\upsilon +\mu )E(t_{j-1})](t_{j}-t_{j-1})+\sigma S(t_{j-1})I(t_{j-1})\\ & \cdot (B(t_{j})-B(t_{j-1}))=\hat{E}(t_{j})+\sigma S(t_{j-1})I(t_{j-1})(B(t_{j})-B(t_{j-1}))\\ \tilde{I}(t_{j})= & I(t_{j-1})+[\upsilon E(t_{j-1})-(\mu +\gamma )I(t_{j-1})](t_{j}-t_{j-1})=\hat{I}(t_{j})\\ \tilde{R}(t_{j})= & R(t_{j-1})+[\gamma I(t_{j-1})-\mu R(t_{j-1})](t_{j}-t_{j-1})=\hat{R}(t_{j})\text{,} \end{array}$$ (31)

where $\hat{S}\left(t_{j}\right)$, $\hat{E}\left(t_{j}\right)$, $\hat{I}\left(t_{j}\right)$ and $\hat{R}\left(t_{j}\right)$ are the solutions of the deterministic system 30, in this case, approximated by Euler Method. We want to find the value of *σ* such as *X* is minimum, where *X* is

$$\begin{array}{c} X=\sum_{j=0}^{n}({\left(\tilde{S}\left(t_{j}\right)-S\left(t_{j}\right)\right)}^{2}+{\left(\tilde{E}\left(t_{j}\right)-E\left(t_{j}\right)\right)}^{2}+{\left(\tilde{I}\left(t_{j}\right)-I\left(t_{j}\right)\right)}^{2}+{\left(\tilde{R}\left(t_{j}\right)-R\left(t_{j}\right)\right)}^{2})\text{.}\; \end{array}$$ (32)

As we do not have the data *S*(*t*_*j*_) and *E*(*t*_*j*_), we take $S(t_{j})\approx \overset{˘}{S}(t_{j})$ and $E(t_{j})\approx \overset{˘}{E}(t_{j})$, values calculated using Λ, *υ*, *μ*, *I*(*t*_*j*_) and *R*(*t*_*j*_). For finding the minimum *σ*, we find ∂*X*/∂*σ* given by

$$\begin{array}{c} \begin{array}{cc} \frac{\partial X}{\partial \sigma }= & -2\sum_{j=1}^{n+1}(\hat{S}\left(t_{j}\right)-\sigma \overset{˘}{S}(t_{j-1})I(t_{j-1}){△}_{B_{j}}-\overset{˘}{S}\left(t_{j}\right))\overset{˘}{S}\left(t_{j-1}\right)I\left(t_{j-1}\right){△}_{B_{j}}\\ & +2\sum_{j=1}^{n+1}(\hat{E}\left(t_{j}\right)+\sigma \overset{˘}{S}(t_{j-1})I(t_{j-1}){△}_{B_{j}}-\overset{˘}{E}\left(t_{j}\right))\overset{˘}{S}\left(t_{j-1}\right)I\left(t_{j-1}\right){△}_{B_{j}}=0\text{,} \end{array} \end{array}$$ (33)

taking △*t* = *t*_*j*_ − *t*_*j*−1_ and ${△}_{B_{j}}=B(t_{j})-B(t_{j-1})$ for all *j* = 1, …, *n*. We have that

$$\begin{array}{c} \begin{array}{cc} 2\sigma \sum_{j=1}^{n+1}{\overset{˘}{S}}^{2}(t_{j-1})I^{2}(t_{j-1}){△}_{B_{j}}^{2}= & \sum_{j=1}^{n+1}\left(\hat{S}\left(t_{j}\right)-\overset{˘}{S}\left(t_{j}\right)\right)\overset{˘}{S}\left(t_{j-1}\right)I\left(t_{j-1}\right){△}_{B_{j}}\\ & +\sum_{j=1}^{n+1}\left(\overset{˘}{E}\left(t_{j}\right)-\hat{E}\left(t_{j}\right)\right)\overset{˘}{S}\left(t_{j-1}\right)I\left(t_{j-1}\right){△}_{B_{j}}\text{,} \end{array} \end{array}$$ (34)

for △_*B*_ ≠ 0. Therefore, the estimator of *σ* is equal to

$$\begin{array}{c} \hat{\sigma }=\frac{\sum_{j=1}^{n+1}\left(\hat{S}\left(t_{j}\right)-\overset{˘}{S}\left(t_{j}\right)+\overset{˘}{E}\left(t_{j}\right)-\hat{E}\left(t_{j}\right)\right)\overset{˘}{S}\left(t_{j-1}\right)I\left(t_{j-1}\right){△}_{B_{j}}}{2\sum_{j=1}^{n+1}{\overset{˘}{S}}^{2}(t_{j-1})I^{2}(t_{j-1}){△}_{B_{j}}^{2}}\text{,} \end{array}$$ (35)

where ${△}_{B_{j}}$ can be a random number generated of a distribution *N*(0, △*t*). We have that $\partial^{2}X/\partial \sigma^{2}=4\sum_{j=1}^{n+1}{\overset{˘}{S}}^{2}(t_{j-1})I^{2}(t_{j-1}){△}_{B_{j}}^{2}>0$, therefore $\hat{\sigma }$ does *X* minimum. However, the values of ${△}_{B_{j}}$ values can significantly change which is why we will take ${△}_{B_{j}}\approx \overset{®}{x}$ where $\overset{®}{x}$ is the mean of 10,000 random values of a distribution *N*(0, △*t*).

Worth noting that initially, we do not know the values of *β* and *γ*. Therefore these are estimated using the Eqs 15 and 17, respectively. There by $\hat{S}(t_{j})$, $\hat{E}(t_{j})$, $\hat{I}(t_{j})$ and $\hat{R}(t_{j})$ are the solutions of the system 1 with $\beta \approx \hat{\beta }$ and $\gamma \approx \hat{\gamma }$, which we establish using the function ode from deSolve package in R.

**Fig 22:**
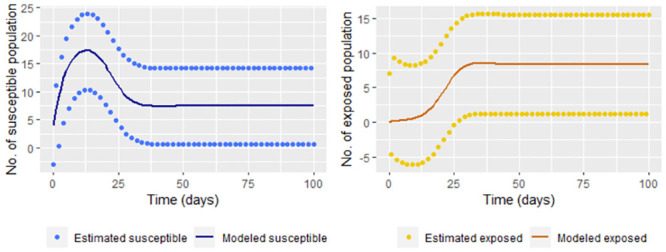
Five paths of the system 31 (Stochastic paths) with Λ = 4, *υ* = 0.1, *μ* = 0.2, $\hat{\beta }$, $\hat{\gamma }$ and $\hat{\sigma }$ (using 15, 17 and 35) calculated for the smoothed simulated data in the steps 1 and 2, $\overset{˘}{S}(t_{j})$ and $\overset{˘}{E}(t_{j})$. In this figure also are graphed the solution of the deterministic system 1 (Deterministic solution) with Λ, *υ* and *μ*, *β*, and *γ* previously given and the simulated data in the steps 1 and 2 (Infected and Recovered data), $\overset{˘}{S}(t_{j})$ (Estimated susceptible) and $\overset{˘}{E}(t_{j})$ (Estimated exposed). $\overset{˘}{S}(t_{j})$ and $\overset{˘}{E}(t_{j})$ are calculated for the smoothed simulated data.

Before estimating the parameters, we suggest smoothing the values of *I*(*t*_*j*_) and *R*(*t*_*j*_) (comparing Figs 11 and 12), which we do for the simulated data in the steps 1 and 2. With it, we have reasonable values of $\overset{˘}{S}(t_{j})$ and $\overset{˘}{E}(t_{j})$ (Eq 11) to calculate $\hat{\sigma }$. We use from day 7 to the last day for estimating *σ* due to $\overset{˘}{E}(t_{j})≉E(t_{j})$ (Fig 10). In the Fig 22 we graph five paths for $\tilde{S}\left(t_{j}\right)$, $\tilde{E}\left(t_{j}\right)$, $\tilde{I}\left(t_{j}\right)$ and $\tilde{R}\left(t_{j}\right)$ (Eq 31) join the data simulated in the steps 1 and 2, $\overset{˘}{S}(t_{j})$ and $\overset{˘}{E}(t_{j})$ calculated of the smoothed simulated data. We obtained $\hat{\sigma }=0.07527228$, which implies a great variability between the paths as can be observed in Fig 22.

On the Eq 31 the values of $\hat{S}(t_{j})$, $\hat{E}(t_{j})$, $\hat{I}(t_{j})$ and $\hat{R}(t_{j})$ are the solutions of the deterministic system 1 for COVID-19 data from Bogota. Given that we had a bad approximation by estimating using least squares (Table 3), we decided to take $\hat{S}(t_{j})$, $\hat{E}(t_{j})$, $\hat{I}(t_{j})$ and $\hat{R}(t_{j})$ by

$$\begin{array}{c} \underline{S}\left(t_{j}\right)=N\left(t_{j}\right)-\overset{⌢}{E}\left(t_{j}\right)-I\left(t_{j}\right)-\overset{⌢}{R}\left(t_{j}\right),\;\underline{E}\left(t_{j}\right)=\overset{⌢}{E}\left(t_{j}\right),\;\underline{I}\left(t_{j}\right)=\overset{⌢}{I}\left(t_{j}\right)\;\text{and}\;\underline{R}\left(t_{j}\right)=\overset{⌢}{R}\left(t_{j}\right); \end{array}$$ (36)

These are the updated data estimations graphed in Fig 18, except for $\underline{S}(t_{j})$ whose estimations are not good using this approach (see Fig 18). It is important to say that $\underline{S}(t_{j})$, $\underline{E}(t_{j})$, $\underline{I}(t_{j})$ and $\underline{R}(t_{j})$ are calculated from *υ* = 1/5.2, *μ* = 4/1000, Λ = 73660 and smoothed infected and recovered COVID-19 data (which we note as *I*(*t*_*j*_) and *R*(*t*_*j*_), respectively). Thus we estimate the volatility parameter as

$$\hat{\sigma }=\frac{\begin{array}{c} \sum_{j=1}^{n+1}(\underline{S}(t_{j})-\overset{˘}{S}(t_{j})+\overset{˘}{E}(t_{j})-\underline{E}(t_{j}))\overset{˘}{S}(t_{j-1})I(t_{j-1}){△}_{B_{j}} \end{array}}{\begin{array}{c} 2\sum_{j=1}^{n+1}{\overset{˘}{S}}^{2}\left(t_{j-1}\right)I^{2}\left(t_{j-1}\right){△}_{B_{j}}^{2} \end{array}}=6.756712\times 10^{-23}$$

Which is negligible by being too near to zero. This is why $\underline{S}(t_{j}),\underline{E}(t_{j})$ are practically equal to $\overset{˘}{S}(t_{j}),\overset{˘}{E}(t_{j})$, respectively (see Figs 18 and 19). For this reason, we take $\hat{S}(t_{j})$, $\hat{E}(t_{j})$, $\hat{I}(t_{j})$ and $\hat{R}(t_{j})$, respectively by $\hat{S}(t_{j})$, $\hat{E}(t_{j})$, $\hat{I}(t_{j})$ and $\hat{R}(t_{j})$ defined as in the Eq 28. We have $\hat{\sigma }$ given by

$$\begin{array}{c} \hat{\sigma }=\frac{\sum_{j=1}^{n+1}(\overset{⌢}{S}\left(t_{j}\right)-\overset{˘}{S}\left(t_{j}\right)+\overset{˘}{E}\left(t_{j}\right)-\overset{⌢}{E}\left(t_{j}\right))\overset{˘}{S}\left(t_{j-1}\right)I\left(t_{j-1}\right){△}_{B_{j}}}{2\sum_{j=1}^{n+1}{\overset{˘}{S}}^{2}(t_{j-1})I^{2}(t_{j-1}){△}_{B_{j}}^{2}}=7.193334\times 10^{-7} \end{array}$$ (37)

**Fig 23:**
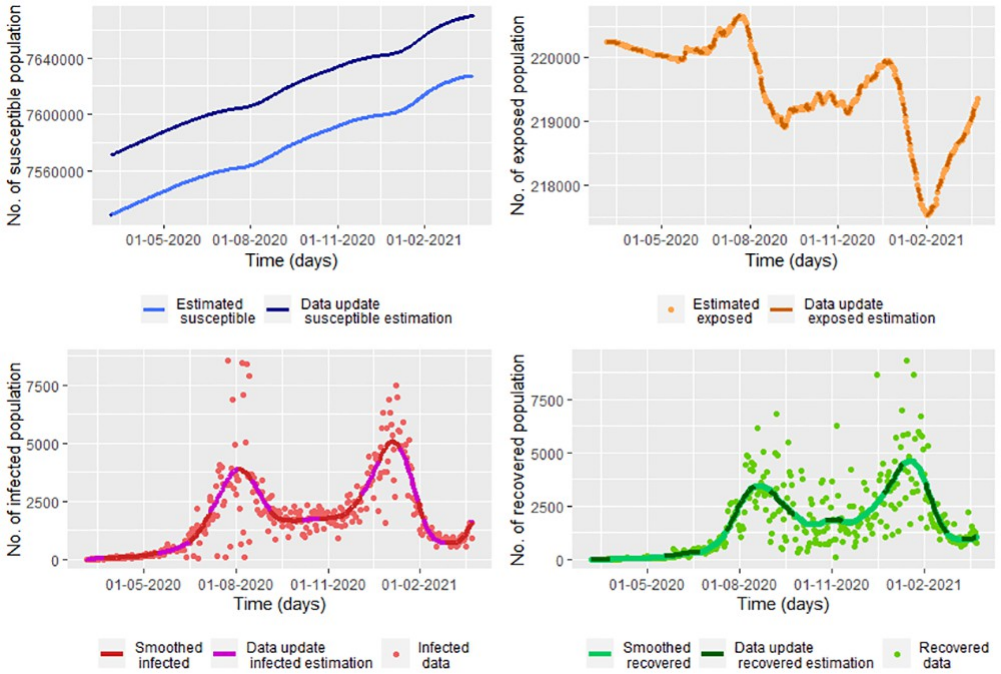
Five paths of the system 38 for susceptible and exposed population. We take Λ = 73660, *μ* = 0.004, ${\hat{\beta }}_{j}$, ${\hat{\upsilon }}_{j}$ and ${\hat{\gamma }}_{j}$ and $\hat{\sigma }$ (using 27, 26, 23 and 37) calculated for the smoothed COVID-19 data from Bogota, $\overset{˘}{S}(t_{j})$ and $\overset{˘}{E}(t_{j})$. $\overset{˘}{S}(t_{j})$ and $\overset{˘}{E}(t_{j})$ are calculated for the smoothed data.

In Fig 23, we graph the solutions for the susceptible and exposed population given by $\underline{S}(t_{j})$ and $\overset{⌢}{E}(t_{j})$ defined as in the Eq 28, join with the stochastic paths under the stochastic model 4 (system 38). Note that we take $\underline{S}(t_{j})$ instead of $\overset{⌢}{S}(t_{j})$ due to with $\overset{⌢}{S}(t_{j})$ there is not a good fit of $\overset{˘}{S}(t_{j})$ (upper left Fig 18). In the Fig 23 the maximum error of prediction, taking the distance between the estimated susceptible population given by $\overset{˘}{S}(t_{j})$ and the paths of the stochastic system 38, for the susceptible population is approximately 77,794, and the maximum prediction error for the exposed population is approximately 60,153.

$$\begin{array}{c} \begin{array}{c} \text{Model}\;\text{4:}\;\\ \tilde{S}(t_{j})=\underline{S}(t_{j})-\hat{\sigma }\overset{˘}{S}(t_{j-1})I(t_{j-1})(B(t_{j})-B(t_{j-1}))\\ \tilde{E}(t_{j})=\overset{⌢}{E}(t_{j})+\hat{\sigma }\overset{˘}{S}(t_{j-1})I(t_{j-1})(B(t_{j})-B(t_{j-1}))\\ \tilde{I}(t_{j})=\overset{⌢}{I}(t_{j})\text{,}\;\tilde{R}(t_{j})=\overset{⌢}{R}(t_{j})\text{.} \end{array} \end{array}$$ (38)

**Fig 24:**
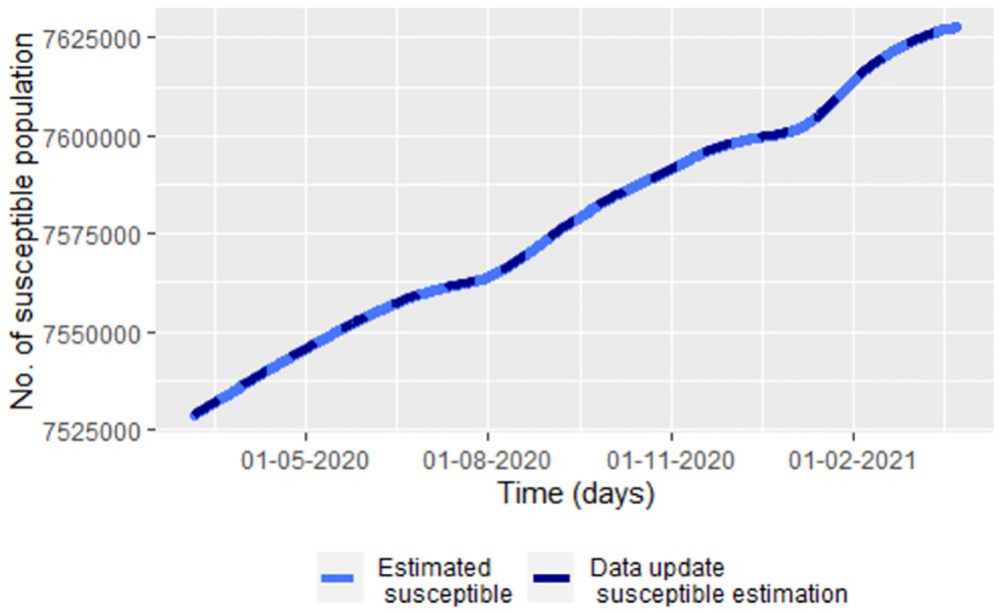
Five paths of $\tilde{I}(t_{j})=N(t_{j})-\tilde{S}\left(t_{j}\right)-\tilde{E}\left(t_{j}\right)-\tilde{R}\left(t_{j}\right)$ and $\tilde{R}(t_{j})=N(t_{j})-\tilde{S}\left(t_{j}\right)-\tilde{E}\left(t_{j}\right)-\tilde{I}\left(t_{j}\right)$ with $\tilde{S}\left(t_{j}\right)$ and $\tilde{E}\left(t_{j}\right)$ from the system 38 for infected and recovered population. We take Λ = 73660, *μ* = 0.004, ${\hat{\beta }}_{j}$, ${\hat{\upsilon }}_{j}$ and ${\hat{\gamma }}_{j}$ and $\hat{\sigma }$ (using 27, 26, 23 and 37) calculated for the smoothed COVID-19 data from Bogota, $\overset{˘}{S}(t_{j})$ and $\overset{˘}{E}(t_{j})$. $\overset{˘}{S}(t_{j})$ and $\overset{˘}{E}(t_{j})$ are calculated for the smoothed data.

In the equation, 38, we have a deterministic graph for the infected and recovered population. So, there is no error prediction. In this way, we propose taking $\tilde{I}(t_{j})=N(t_{j})-\tilde{S}\left(t_{j}\right)-\tilde{E}\left(t_{j}\right)-\tilde{R}\left(t_{j}\right)$ and $\tilde{R}(t_{j})=N(t_{j})-\tilde{S}\left(t_{j}\right)-\tilde{E}\left(t_{j}\right)-\tilde{I}\left(t_{j}\right)$ for having that error, whose paths graph in the Fig 24. Maximum error of prediction for the infected population for the infected population is approximately 502, and the maximum prediction error for the recovered population is the same. In this case, the distance between the smoothed infected COVID-19 data from Bogota and the paths of $N(t_{j})-\tilde{S}\left(t_{j}\right)-\tilde{E}\left(t_{j}\right)-\tilde{R}\left(t_{j}\right)$ with $\tilde{S}\left(t_{j}\right)-\tilde{E}\left(t_{j}\right)$ from the system 38

**Fig 25:**
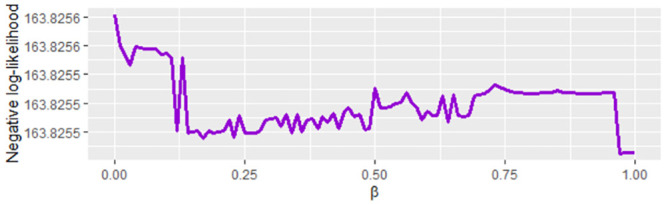
Fitted values for each population concerning the residuals according to the model 39.

We study the homoscedasticity according to the graphs of fitted values of the populations concerning the residuals, which correspond to the Fig 25. We do a residual analysis under the regression focus using the model given by the system 39. In Fig 25, we have heteroscedasticity for all the populations under the regression model provided by the system 39, where it is assumed that the residuals for each population are independent.

$$\begin{array}{c} \begin{array}{c} \overset{˘}{S}(t_{j})=\overset{⌢}{S}(t_{j})+\epsilon_{s},\epsilon_{s}∼N\left(0,\sigma_{s}^{2}\right)\\ \overset{˘}{E}(t_{j})=\overset{⌢}{E}(t_{j})+\epsilon_{e},\epsilon_{e}∼N\left(0,\sigma_{e}^{2}\right)\\ I(t_{j})=\tilde{I}(t_{j})+\epsilon_{i},\epsilon_{i}∼N\left(0,\sigma_{i}^{2}\right)\\ R(t_{j})=\tilde{R}(t_{j})+\epsilon_{r},\epsilon_{r}∼N\left(0,\sigma_{r}^{2}\right) \end{array} \end{array}$$ (39)

Table 6 shows the p-values obtained for the Shapiro-Wilks test for normality for all the residuals given the paths under the model 39. Note that all the p-values are more significant than 0.05, so we conclude with a confidence of 95% that the residuals do not present normal distribution.

**Table 6:** p-values for each one of the paths under the model 39 under the Shapiro-Wilks test for normality.

Population	c p-value for the 1st path	c p-value for the 2nd path	c p-value for the 3rd path	c p-value for the 4th path	c p-value for the 5th path
Susceptible	1.892e-13	6.565e-15	1.624e-14	6.224e-18	1.671e-14
Exposed	1.789e-13	6.259e-15	1.631e-14	5.988e-18	1.739e-14
Infected	2.207e-24	2.207e-24	2.207e-24	2.207e-24	2.207e-24
Recovered	6.344e-18	6.344e-18	6.344e-18	6.344e-18	6.344e-18

## Conclusions

This paper proposes estimation methods using a data update approach for the COVID-19 data in Bogota. Our methods of estimation based on recovered as infected data: (1) a method based on the likelihood function with variance given by the Eq 6 (model 2); (2) a method based on ordinary least squares on the infected and recovered data given by the Eq 7; and (3) a data update method based on the recovered, infected and exposed data; given by the Eqs 23, 26 and 27 (model 3). Method (1) has the issue that other populations can be overestimated. In contrast, the other methods base their estimation on first approximating the susceptible and exposed population using some known parameters, which are Λ, *μ*, and *υ*. In particular, method (1) could be convenient for the initial phase of increasing the disease; and when there is not enough knowledge about, for instance, how fast the epidemic spreads. However, their estimators may change significantly by small changes in the initial conditions. Method (2) does not always fit the data, and depending on the known parameters, this method may present a different scale in their solutions. It is worth noting that although model (2) has a good fit, we consider that the model methodology may not determine the exceeded cases.

The proposed models capture the data from Bogotá city well. However, it is potentially limited by the lack of prevalence data corresponding to the registration in individuals who present specific variants of the disease over a period of time, including susceptible, exposed, and vaccinated sub-populations, and also data of asymptomatic infected. Also, we have not considered age-structured models in social behaviors and transmission rates. We create the data update approach for a good fit. Under the update data method, we can think of the parameters as functions depending on the time or a time-series model of the ARIMA process. However, the parameter *υ* must be newly estimated under this. We concluded that the best method for fitting an epidemic mathematical model to infected and recovered COVID-19 data from Bogota, D.C. is by using the model 3 and taking the susceptible population as $\underline{S}(t_{j})$. If we wished to make trusted bands, we could estimate the parameters by the Eqs 23, 26 and 27 and approximate by a time-series process for predicting the behavior of an epidemic based on the infected and recovered data. This paper has established some methodologies of parameter estimation on models based on ordinary differential systems and stochastic differential systems to one of the simplest models: the SEIR model. In future research, we expect to develop the data update approach to models with more compartments, including symptomatic, hospitalized, and dead for the COVID population.

## Supporting information

S1 File (PDF)
