# Supplementary material for: An updated estimation approach for SEIR models with stochastic perturbations: Application to COVID-19 data in Bogotá
Source: PLoS One. 2023 Aug 21;18(8):e0285624. doi: 10.1371/journal.pone.0285624 (PMC10441809; doi:10.1371/journal.pone.0285624)
Supplement: S1 File — (PDF) [file pone.0285624.s001.pdf]

# Annexes

## A List of variables and models

In the following tables, we resume the information, the list of the variables used in each model 7 and proposed models 8.

| Variables | Description | Eq. |
|---|---|---|
| $\phi_1(t_j), \phi_2(t_j), \phi_3(t_j), \phi_4(t_j)$ | Regression functions corresponding to the solutions of the system 1 for the susceptible, exposed, infected and recovered population, respectively. | 2 |
| $\hat{S}(t_i), \hat{E}(t_i), \hat{I}(t_i), \hat{R}(t_i)$ | Solutions for each population of the deterministic system 1 by the Euler method using all of types of population data. | 8 |
| $\breve{S}(t_i), \breve{E}(t_i)$ | Estimated susceptible and exposed population by solving the system 8 using $\upsilon$, infected and recovered data. | 11 |
| $\overset{\smile}{S}(t_j), \overset{\smile}{E}(t_j)$ | Estimated susceptible and exposed population by solving the system 8 using $\upsilon$, $\gamma$ and infected data. | 20 |
| $\overset{\angle}{S}(t_j), \overset{\angle}{E}(t_j)$ | Estimated susceptible and exposed population by solving the system 8 using $\upsilon$, $\gamma$, infected data and estimated recovered data ($\hat{R}(t_i)$). | 22 |
| $\overset{*}{S}(t_j), \overset{*}{E}(t_j), \overset{*}{I}(t_j), \overset{*}{R}(t_j)$ | Approximations of the solutions for each population of the deterministic system 1 by the Euler method using estimated susceptible and exposed population ($\breve{S}(t_i), \breve{E}(t_i)$), infected and recovered data, and $\gamma$ and $\beta$ given by 23 and 24, respectively. | 25 |
| $\overset{\frown}{S}(t_j), \overset{\frown}{E}(t_j), \overset{\frown}{I}(t_j), \overset{\frown}{R}(t_j)$ | Approximations of the solutions for each population of the deterministic system 1 by the Euler method using estimated susceptible and exposed population ($\breve{S}(t_i), \breve{E}(t_i)$), infected and recovered data, and $\gamma$, $\upsilon$ and $\beta$ given by 23, 26 and 15, respectively. | 28 |
| $\tilde{S}(t_j), \tilde{E}(t_j), \tilde{I}(t_j), \tilde{R}(t_j)$ | Approximations of the solutions of the solutions for each population of the stochastic system 30 by the Euler-Maruyama method using all of types of population data. | 31 |
| $\underline{S}(t_j)$ | Solution for the susceptible population of the deterministic system 1 given by $\underline{S}(t_j) = N(t_j) - \overset{\frown}{E}(t_j) - \overset{\frown}{I}(t_j) - \overset{\frown}{R}(t_j)$, where $N(t_j)$ is the total population on the time $t_j$. | 36 |

**Table 7.** List of variables used in this paper.

| Model | Description | Eq. |
|---|---|---|
| Model 1 | Non linear regression model where the regression functions are the solutions of the system 1 given by the function `ode` from the package `deSolve` in `R`. The estimation method is based on maximum likelihood with variance on only the infected data. | 4 |
| Model 2 | Non linear regression model where the regression functions are the solutions of the system 1 given by the function `ode` from the package `deSolve` in `R`. The estimation method is based on maximum likelihood with variance on the infected and recovered data. | 5 |
| Model 3 | This system are the approximations to the solutions of the deterministic system 1, by the Euler method using estimated susceptible and exposed population $(\breve{S}(t_i), \breve{E}(t_i))$, infected and recovered data, and $\gamma$, $\upsilon$ and $\beta$ estimated by data update approach, that is, by 23, 26 and 15, respectively. | 28 |
| Model 4 | This system are the approximations to the solutions of the stochastic system 31, by the Euler-Maruyama method using estimated susceptible population by $\underline{S}(t_j)$, estimated exposed population by $\widehat{E}(t_i)$, infected and recovered data, and $\sigma$ estimated by 37. | 38 |

**Table 8.** Models proposed for each section.
